# TPM, FPKM, or Normalized Counts? A Comparative Study of Quantification Measures for the Analysis of RNA-seq Data from the NCI Patient-Derived Models Repository

**DOI:** 10.1186/s12967-021-02936-w

**Published:** 2021-06-22

**Authors:** Yingdong Zhao, Ming-Chung Li, Mariam M. Konaté, Li Chen, Biswajit Das, Chris Karlovich, P. Mickey Williams, Yvonne A. Evrard, James H. Doroshow, Lisa M. McShane

**Affiliations:** 1 Biometric Research Program, Division of Cancer Treatment and Diagnosis, National Cancer Institute, Rockville, MD USA; 2 Leidos Biomedical Research, Inc., Frederick National Laboratory for Cancer Research, Frederick, MD USA; 3 Division of Cancer Treatment and Diagnosis, National Cancer Institute, Bethesda, MD USA

**Keywords:** RNA sequencing, Quantification measures, Normalization, TPM, FPKM, Count, RSEM, Patient derived xenograft models, DESeq2, TMM

## Abstract

**Background:**

In order to correctly decode phenotypic information from RNA-sequencing (RNA-seq) data, careful selection of the RNA-seq quantification measure is critical for inter-sample comparisons and for downstream analyses, such as differential gene expression between two or more conditions. Several methods have been proposed and continue to be used. However, a consensus has not been reached regarding the best gene expression quantification method for RNA-seq data analysis.

**Methods:**

In the present study, we used replicate samples from each of 20 patient-derived xenograft (PDX) models spanning 15 tumor types, for a total of 61 human tumor xenograft samples available through the NCI patient-derived model repository (PDMR). We compared the reproducibility across replicate samples based on TPM (transcripts per million), FPKM (fragments per kilobase of transcript per million fragments mapped), and normalized counts using coefficient of variation, intraclass correlation coefficient, and cluster analysis.

**Results:**

Our results revealed that hierarchical clustering on normalized count data tended to group replicate samples from the same PDX model together more accurately than TPM and FPKM data. Furthermore, normalized count data were observed to have the lowest median coefficient of variation (CV), and highest intraclass correlation (ICC) values across all replicate samples from the same model and for the same gene across all PDX models compared to TPM and FPKM data.

**Conclusion:**

We provided compelling evidence for a preferred quantification measure to conduct downstream analyses of PDX RNA-seq data. To our knowledge, this is the first comparative study of RNA-seq data quantification measures conducted on PDX models, which are known to be inherently more variable than cell line models. Our findings are consistent with what others have shown for human tumors and cell lines and add further support to the thesis that normalized counts are the best choice for the analysis of RNA-seq data across samples.

**Supplementary Information:**

The online version contains supplementary material available at 10.1186/s12967-021-02936-w.

## Background

RNA-sequencing (RNA-seq) has replaced gene expression microarrays as the most popular method for transcriptome profiling [1, 2]. Various computational tools have been developed for RNA-seq data quantification and analysis, sharing a similar workflow structure, but with some notable differences in certain processing steps [3, 4]. Starting from a FASTQ file containing sequence reads and corresponding quality scores, the sequence reads can be mapped and aligned to a reference genome using algorithms such as TopHat2 and/or STAR read aligner. Gene counts are then generated from the resulting SAM or BAM file using tools such as SAMtools and HTSeq. This process is time consuming and yields gene-level counts only. Because alternative splicing creates multiple structurally-distinct transcripts of the same gene that may produce different phenotypes, several tools have been developed for RNA-seq isoform quantification such as Salmon_aln, eXpress, RSEM, and TIGAR2, which all require transcriptome-mapping BAM files [5]. In contrast to the aforementioned alignment-based methods, transcript quantification tools Salmon, Sailfish, and kallisto were designed to boost processing speed and to decrease memory and disk usage by bypassing the creation and storage of BAM files [6–8]. This approach is particularly useful for the discovery of novel transcripts, when sequencing poorly annotated transcriptomes, and to detect lowly expressed genes [9]. Raw read counts cannot be used to compare expression levels between samples due to the need to account for differences in transcript length, total number of reads per samples, and sequencing biases [4]. Therefore, RNA-seq isoform quantification software summarize transcript expression levels either as TPM (transcript per million), RPKM (reads per kilobase of transcript per million reads mapped), or FPKM (fragments per kilobase of transcript per million reads mapped); all three measures account for sequencing depth and feature length [4].

Because of the nature of the quantification measures and embedded implicit normalization process, TPM, RPKM, and FPKM expression levels are suitable for the comparison of RNA transcript expression within a single sample. However, none of these measures can be used universally for cross-sample comparisons and downstream analyses such as the determination of differentially expressed genes between two or more biological states. Issues arise, especially in the case of lowly expressed genes, when attempts are made to correct for gene length differences [9]. In a comprehensive evaluation of normalization methods for Illumina high-throughput RNA-seq data analysis, Dillies et al*.* [9] concluded that total gene counts and RPKM were not recommended quantifications for use in downstream differential expression analysis. Only DESeq2 and TMM normalization methods were shown to produce quantifications robust to the presence of different library sizes and widely different library compositions. Conesa et al*.* [4] conducted a survey of best practices for RNA-seq data analysis and indicated that RPKM, FPKM, and TPM methods normalize away the most important factor for comparing samples, which is sequencing depth, whether directly or by accounting for the number of transcripts, which can differ significantly between samples. RPKM, FPKM, and TPM tend to perform poorly when transcript distributions differ between samples. Highly expressed features in certain samples can skew the quantitative measure distribution and adversely affect normalization, leading to the spurious identification of differentially expressed genes. Zhao et al*.* [10] recently reported the misuse of RPKM and TPM normalization when comparing data across samples and sequencing protocols. However, due to the lack of experimental data generated from different types of replicates to further validate their recommendation, consensus regarding which RNA-seq quantification measure should be used for cross-sample comparison seems not to have been reached by the scientific community. Many recent peer-reviewed articles, as well as publicly-available databases and websites, are still using TPM or RPKM/FPKM for pooled data analyses, cross-sample comparisons, and differential expression (DE) analysis [11–15]. Furthermore, some researchers have attempted to improve comparability of the expression measures by applying certain transformations (e.g., median centering and unit variance scaling, also referred to here as Z-score) or re-normalizing on either TPM or RPKM/FPKM data.

In recent years cancer models developed from patient tumors have come to replace late passage cell lines as the preferred tool in pre-clinical cancer research [16]. The resulting patient-derived xenograft (PDX) models recapitulate most histological and genetic characteristics of their human donor tumor, thus facilitating the prediction of clinical outcomes and the investigation of drug efficacy, biomarker identification, and development of personalized medicine strategies. The National Cancer Institute (NCI) is developing a national repository of Patient-Derived Models (PDMs) comprised of hundreds of patient-derived xenograft (PDX) models spanning a wide variety of tumor types. The publicly-accessible database, NCI PDMR (https://pdmr.cancer.gov/), provides clinical annotations as well as molecular characterization information, whole exome sequencing, and RNA-seq data for early-passage PDXs, and if available, for originator patient specimens, to aid in selection of the best model for the investigation of a specific research question.

Here we report on our evaluation of TPM, FPKM, and normalized counts on an RNA-seq dataset of PDX models from the NCI PDMR. Our study examined 61 replicate samples belonging to 20 different PDX models originating from patients with different cancer types to determine which quantitative measures should be used to minimize differences between replicate samples, while preserving biologically meaningful expression differences between genes and across PDX models.

## Methods

### Sample selection and RNA-seq data acquisition

We focused on early-passage PDXs due to the similarity of their genomic and transcriptional profiles to those of the original tumor [17]. RNA-seq data for 61 early-passage (passage 0, 1, and 2) tumor xenografts of human origin belonging to 20 distinct patient-derived xenograft (PDX) models were downloaded from the publicly-accessible NCI PDMR website (https://pdmr.cancer.gov/). In this paper, we used the term “replicate” to denote samples from the same tumor implanted into different mice (i.e., biological replicates). Of the 20 PDX models, 19 had three replicate samples from the same passage with available RNA-seq data, while the remaining model had four replicate samples from the same passage. The 20 PDX models covered 15 different cancer subtypes (Additional file 1: Table S1).

The detailed standing operating procedures for the RNA-seq library preparation and data processing can be found in the SOP section of the NCI PDMR website (https://pdmr.cancer.gov/sops/). Briefly, the samples were sequenced on the Illumina HiSeq Sequencing platform. FASTQ files were generated with bcl2fastq (version: 2.17.1.14, Illumina). Adaptors were trimmed within this process using the default cutoff of the adapter-stringency option. PDX mouse reads were bioinformatically removed from the raw FASTQ files using bbsplit (bbtools v37.36). The fastq files were mapped to the human transcriptome based on exon models from hg19 using Bowtie2 (version 2.2.6). The resulting SAM files were converted to BAM format using samtools, and the transcriptomic coordinates from the BAM file were converted to the corresponding genomic (hg19) coordinates using RSEM (version 1.2.31). Gene and transcript level quantification were also performed with RSEM (version 1.2.31). In our comparative study, we focused on the gene level output files, which contained the TPM, FPKM, expected counts, and effective length for 28,109 genes.

### Quantification and normalization methods

The aim of the present study was to compare the performance of different RNA-seq gene expression quantification measures for downstream analysis. All gene expression measures included in our study are defined below.

#### RPKM and FPKM

The measure RPKM (reads per kilobase of exon per million reads mapped) was devised as a within-sample normalization method; as such, it is suitable to compare gene expression levels within a single sample, rescaled to correct for both library size and gene length [1].

FPKM stands for fragments per kilobase of exon per million mapped fragments. It is analogous to RPKM and is used specifically in paired-end RNA-seq experiments [17]. The calculation of RPKM or FPKM for gene *i* uses the following formula:

$$RPKM_{i} ~{\text{or}}~FPKM_{i} = \frac{{q_{i} }}{{\frac{{l_{i} }}{{10^{3} }}*\frac{{\mathop \sum \nolimits_{j} q_{j} }}{{10^{6} }}}} = \frac{{q_{i} }}{{l_{i} *\mathop \sum \nolimits_{j} q_{j} }}*10^{9}$$

where $q_{i}$ are raw read or fragment counts, $l_{i}$ is feature (i.e., gene or transcript) length, and $\mathop \sum \limits_{j} q_{j}$ corresponds to the total number of mapped reads or fragments. The RSEM output files containing RNA-seq data for the selected samples downloaded from the NCI PDMR include both FPKM and TPM expression values.

#### TPM

TPM was introduced in an attempt to facilitate comparisons across samples. TPM stands for transcript per million, and the sum of all TPM values is the same in all samples, such that a TPM value represents a relative expression level that, in principle, should be comparable between samples [18].

$$TPM_{i} = \frac{{q_{i} /l_{i} }}{{\mathop \sum \nolimits_{j} \left( {q_{j} /l_{j} } \right)}}*10^{6}$$

where *q*_*i*_ denotes reads mapped to transcript, *l*_*i*_ is the transcript length, and $\mathop \sum \limits_{j} (q_{j} /l_{j} )$ corresponds to the sum of mapped reads to transcript normalized by transcript length.

The TPM measure can easily be converted to FPKM: $TPM_{i} = \left( {\frac{{FPKM_{i} }}{{\mathop \sum \nolimits_{j} FPKM_{j} }}} \right)*10^{6} .$

#### Count normalization methods

The R package tximport was used to prepare gene level count data from RSEM output files [19]. Subsequently, normalized count data were derived using the DESeq2 package [20]. The normalization approach used by DESeq2 is to form a “virtual reference sample” by taking the geometric mean of counts over all samples for each gene [20]. Then, DESeq2 normalizes each sample to this virtual reference to get one scaling factor per sample.

TMM stands for a weighted trimmed mean of M values, which are gene-wise log-fold change quantities originally defined by Robinson and Oshlack [21]. Normalization using the TMM method was performed on count data generated from tximport with the ‘tmm’ function in Bioconductor package NOISeq [22]. The TMM normalization method is also implemented in the edgeR package [21].

#### Z-score normalization on TPM-level data

Z-score normalization is considered a centering and variance stabilization method. Z-score on TPM-level data was calculated using the following formula:

$$Z_{{ij}} = \frac{{log_{2} \left( {TPM_{{ij}} + 1} \right) - median\left( {log_{2} \left( {TPM_{i} + 1} \right)} \right)}}{{SD\left( {log_{2} \left( {TPM_{i} + 1} \right)} \right)}}$$

where the indices *i* and *j* stand for gene and sample index, respectively; and SD stands for standard deviation.

### Measures of variation

#### Hierarchical clustering

The R function ‘hclust’ was used for sample clustering based on gene expression matrices. The distance matrix is based on 1 − r, where r is the Pearson correlation coefficient between sample pairs. Ward’s minimum variance method (i.e., linkage method option ‘ward.D2’) was used as the agglomeration method [23, 24]. Euclidean distance metric was also computed to evaluate which measure could more closely align the replicates, in terms of absolute expression measures, for each PDX model.

#### Median CV

The coefficient of variation (CV) was defined as the ratio of the standard deviation to the mean expression of each gene across replicate samples within each of the 20 PDX models. The median CV, as well as the interquartile range, were documented for each PDX model.

#### Intraclass correlation coefficient (ICC)

For each PDX model, an intraclass correlation coefficient, denoted by ICC_g_, was computed to examine the impact of each quantification measure on the variability between genes relative to the total variation (across genes and replicate samples) [24–26].

This analysis was based on a components of variance model:

$$Y_{{ij}} = g_{i} + e_{{ij}}$$

where $Y_{{ij}}$ denotes the log transformed unit of gene *i* in the replicate *j* for a particular model. The error variance component $\sigma _{e}^{2}$ associated with $e_{{ij}}$ (technical error) reflects the reproducibility of the measure. The variance component $\sigma _{g}^{2}$ associated with $g_{i}$ (true gene expression) represents the true gene-to-gene variability.

The intra-class correlation (ICC_g_) for each PDX model is defined as

$$ICC_{g} = \frac{{\sigma _{g}^{2} }}{{\sigma _{g}^{2} + \sigma _{e}^{2} }}$$

and estimated by the following equation defined by Shrout et al*.* [25]:

$$\frac{{MS_{g} - MS_{e} }}{{MS_{g} + \left( {k - 1} \right)MS_{e} }}$$

where $MS_{g}$ is the between-genes mean squares, $MS_{e}$ is the between-samples mean squares, *k* is the number of samples. The ICC_g_, which ranges between 0 and 1, estimates the proportion of the total variance due to the between-gene variance. Larger ICC_g_ values indicate higher similarity (i.e., agreement) between replicate samples while preserving biological differences among genes within a PDX model. Computing an ICC_g_ for each PDX model, as described above, resulted in a set of 20 ICC_g_ values for each quantification method.

Next, in order to evaluate which measure can better preserve true biological differences within the same gene across different PDX models, another version of intraclass correlation, denoted by ICC_m_, was computed for each gene. This metric allowed for examination of the impact of each quantification measure on the variability between PDX models relative to the total variation (across models and replicate samples). This analysis was based on a components of variance model:

$$Y_{{ij}} = m_{i} + e_{{ij}}$$

where $Y_{{ij}}$ denotes the log transformed unit of PDX model *i* in the replicate *j* for a particular gene. For simplicity of notation, gene index was not included in the formula. The error variance component $\sigma _{e}^{2}$ associated with $e_{{ij}}$ (technical error) reflects the reproducibility of the measure. The variance component $\sigma _{m}^{2}$ associated with $m_{i}$ (true gene expression) represents the true model-to-model variability.

The intra-class correlation (ICC_m_) for each gene is defined as

$$ICC_{m} = \frac{{\sigma _{m}^{2} }}{{\sigma _{m}^{2} + \sigma _{e}^{2} }}$$

and estimated by the following equation defined by Shrout et al*.* [25]:

$$\frac{{MS_{m} - MS_{e} }}{{MS_{m} + \left( {k - 1} \right)MS_{e} }}$$

where $MS_{m}$ is the between-models mean squares,$MS_{e}$ is the between-samples mean squares, *k* is the number of samples. The ICC_m_, which ranges between 0 and 1, estimates the proportion of the total variance due to the between-model variance. Larger ICC_m_ values indicate higher similarity (i.e., agreement) between replicate samples. Computing an ICC_m_ for each gene, as described above, resulted in a set of 28,109 ICC_m_ values for each quantification method. A known feature of the ICC estimator used here is that sometimes it could produce negative values when the true ICC is close to zero and sample size is small. For practical purposes, these negative estimates of ICC are considered to be equivalent to ICC ≈ 0.

Model 947758-054-R is the only model that has four replicates, while the other 19 models all have three replicates. For simplicity, the first three replicates of model 947758-054-R were selected to form a uniform data matrix (20 × 3 for each gene) for the calculation of ICC for each gene. The resulting balance in number of replicates allowed for easier calculation of the ICC_g_ and ICC_m_ estimates using the irr R package (version 0.84.1) [25, 26].

### Calculation of percentages of TPM for the top five most abundant genes

To help identify what may cause transcript distribution differences between replicates, we calculated the percentage of TPM for the top five most abundant genes. For each PDX model, the 28,109 genes were first sorted by the sum of TPMs across the replicate samples. The TPM percentages of the top five most abundant genes in each replicate was then calculated as the sum of TPMs corresponding to the top five most abundant genes identified for each model divided by 10^6^.

## Results

### Hierarchical clustering on normalized count data performs the best for grouping replicate samples from the same PDX model

**Fig. 1:**
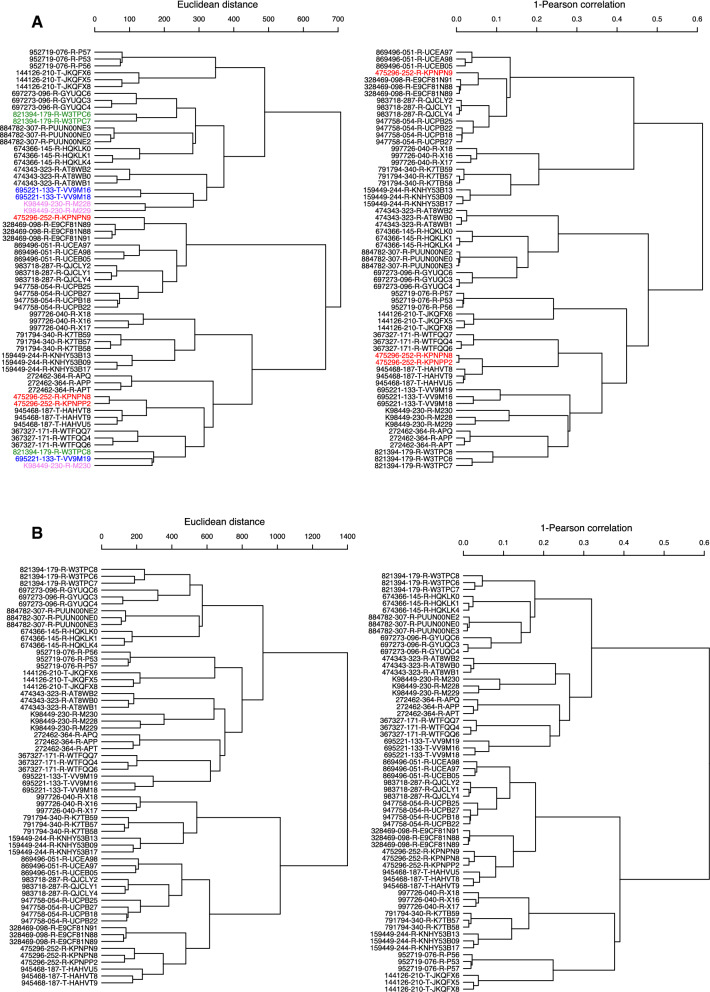
**A** Hierarchical clustering of 61 patient-derived xenograft (PDX) samples using TPM data. **B** Hierarchical clustering of 61 PDX samples using DESeq2 normalized count data. Distance metric 1-Pearson correlation was used to generate the dendrogram in each right panel and Euclidean distance was used for the dendrogram in each left panel. Discordant models are highlighted with different color labels

We performed hierarchical clustering analysis on all 61 samples using different quantification measures, i.e., TPM, FPKM, normalized counts, as well as Z-score normalization on TPM-level data. The pattern of sample clustering differed depending on the gene expression quantification measure used (Fig. 1A, B). Previous studies have shown that for clusters with nearly equal sample sizes, Ward’s method performed significantly better than the other clustering procedures [27–30]. We also tried the “complete” linkage method and found similar patterns to those obtained with Ward linkage for each scenario. In our dataset which is comprised of three or four replicates each for 20 different PDX models, the implementation of different agglomeration methods did not noticeably affect the results.

For clustering based on 1-Pearson correlation distance matrix generated using TPM data (Fig. 1A, right panel), the three samples from PDX model 475296-252-R (rectum) did not cluster together despite being replicate samples originating from the same human tumor. Two of its samples (475296-252-R-KPNPN8 and 475296-252-R-KPNPP2) clustered with a different PDX model from the same cancer type (945468-187-T, rectum), while the third sample (475296-252-R-KPNPN9) clustered with PDX model 328469-098-R (colon). When Euclidean distance was used instead of 1-Pearson correlation as the distance matrix, the performance of the clustering for TPM data was worse. In addition to model 475296-252-R, replicates in another three PDX models, 821394-179-R (Malignant fibrous histiocytoma), 695221-133-T (Melanoma), and K98449-230-R (Glioblastoma), were also not grouped in the same cluster (Fig. 1A, left panel).

When normalized count data using DESeq2 (Fig. 1B) or TMM (Additional file 1: Figure S1A) were used, all replicate samples from the sample PDX model clustered with each other no matter which distance matrix was used, that is, either 1-Peason correlation or Euclidean distance. This was also true when FPKM was used for clustering (Additional file 1: Figure S1B); however, we noticed that for certain models, the maximum distance (1-Pearson correlation) among samples was noticeably larger compared to clustering on DESeq2 or TMM-normalized data (Additional file 1: Figure S2). Table 1 summarizes the number of discordant models while Table 2 lists the maximum height in hierarchical cluster analysis for each data normalization method.

**Table 1:** Number of discordant models in hierarchical cluster analysis under all scenarios

Distance matrix	TPM (Fig. 1A)	CountDEseq2 (Fig. 1B)	CountTMM (Additional file 1: Figure S1A)	FPKM (Additional file 1: Figure S1B)	TPM-Zscore (Additional file 1: Figure S1C)	TPM-TMM (Additional file 1: Figure S3A)
1-Pearson	1/20	0	0	0	1/20	0
Euclidean	4/20	0	0	0	6/20	0

**Table 2:** Maximum height in hierarchical cluster analysis under all scenarios

Distance matrix	TPM (Fig. 1A)	CountDEseq2 (Fig. 1B)	CountTMM (Additional file 1: Figure S1A)	FPKM (Additional file 1: Figure S1B)	TPM-Zscore (Additional file 1: Figure S1C)	TPM-TMM (Additional file 1: Figure S3A)
1-Pearson	0.613	0.091	0.089	0.106	3.152^a^	0.102

^a^Since Ward method is used as the linkage method, the height is not limited to the original scale and can be larger than 2

### Normalized count data has the minimum median CV across replicates from the same PDX model

**Fig. 2:**
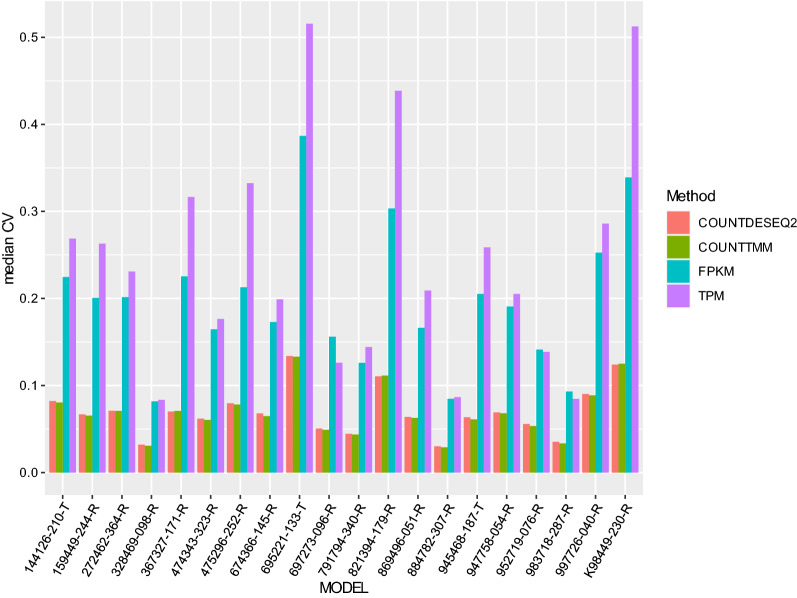
Bar plot of median coefficients of variation (CV) for gene expression levels from replicate samples of each PDX model using different quantification measures

We then calculated the median coefficient of variation (CV) across the replicate samples for each PDX model. Figure 2 displays the median CVs for each model using different quantification measures. Among all PDX models, median CVs from either DESeq2-normalized count data (Fig. 2, red bars) or TMM-normalized data (Fig. 2, green bars) were on par with each other (ranging from 0.05 to 0.15), and were low when compared to median CVs from TPM (Fig. 2, purple bars) or FPKM data (Fig. 2, cyan bars). Among the four different quantification measures, TPM was the worst performer with the largest median CVs (ranging from 0.08 to 0.52), while FPKM also performed worse than normalized count data, but better than TPM in the majority of the models. Overall, normalized count data had the smallest median CVs compared to TPM and FPKM data across replicate samples in all 20 PDX models. Summary statistics on CVs, including the interquartile range, are listed in Additional file 1: Table S2 for different quantitative measures.

### Normalized count data has better ICC values over TPM and FPKM data for all PDX models

Next, we explored which quantitative measure minimized differences between replicate samples, while preserving biologically meaningful expression differences between genes and across PDX models. These assessments were based on the distributions of 20 ICC_g_ and 28,109 ICC_m_ values for each quantification method. Higher ICC values are indicative of better reproducibility between replicates [31].

**Fig. 3:**
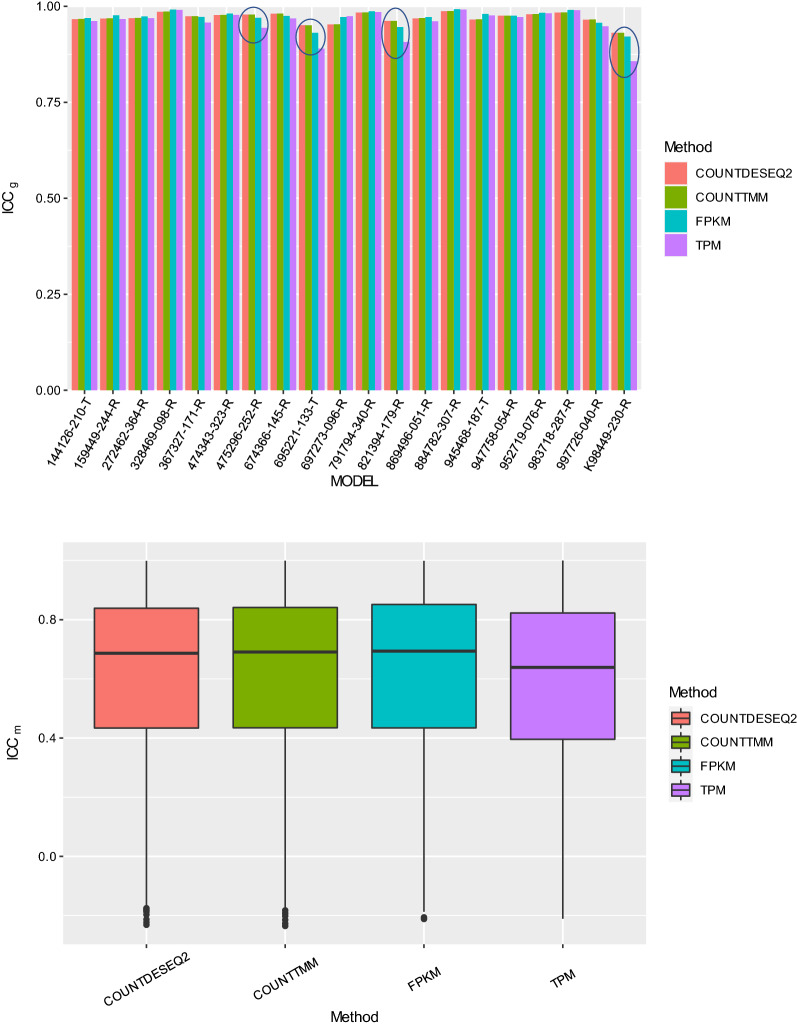
**A** Bar plot of gene intraclass correlation coefficients (ICC_g_) across replicate samples of each PDX model using different quantification measures. **B** Boxplots of model intraclass correlation coefficients (ICC_m_) for gene expression levels from replicate samples across 20 PDX models using different quantification measures

Figure 3A illustrates the comparison of ICC_g_ when using different RNA-seq quantification measures on the 20 PDX models. Although all ICC_g_ values were above 0.85, quantification measures still performed variably in at least four PDX models. Among them, TPM data (Fig. 3A, purple bars) had the lowest ICC_g_ values for PDX models 475296-252-R, 695221-133-T, 821394-179-R, and K98449-230-R [ranges of ICC_g_ in four models was (0.859, 0.944)], while normalized count data using either DESeq2 (Fig. 3A, red bars) or TMM (Fig. 3A, green bars) had the highest ICC_g_ values [ranges of ICC_g_s in four models were (0.931, 0.979) for DESeq2 and (0.931, 0.979) for TMM]. Furthermore, FPKM data had lower ICC_g_ values than DESeq2 and TMM-normalized count data in the above four models. Those four models were the same models identified in hierarchical clustering using Euclidean distance whose replicates did not cluster with each other. These results indicate that the normalized count data were more reproducible across replicate samples, in the sense of having generally higher between-gene variance relative to the total variation (across genes and replicate samples) across PDX models.

We also calculated ICC_m_ for each gene to examine the impact of each quantification measure on both within-model error variance (between replicate samples for the same gene) and between-model variance for each gene (model ICC_m_). Similarly, larger ICC_m_ indicates that the replicate error variance is relatively small compared to the biological differences across PDX models for each gene.

Figure 3B shows the comparison of model ICC_m_ when using different RNA-seq quantification measures on all 28,109 genes. Normalized count from DESeq2 or TMM, as well as FPKM performed similarly well with median ICC_m_ around 0.69, while TPM performed the worst with median ICC_m_ of 0.64. These results indicate that the normalized count data were more reproducible across replicate samples, in the sense of having generally higher between-model variance relative to the total variation (across models and replicate samples) across genes.

### Neither Z-score nor an additional normalization step can resolve the potentially problematic issue of TPM data

We further checked whether Z-score transformation or an additional normalization step would help to resolve the potentially problematic issue of TPM data, especially for PDX model 475296-252-R. We found that even after Z-score normalization of TPM data, the replicate samples for PDX model 475296-252-R remained separated following hierarchical clustering (Additional file 1: Figure S1C, right panel), similar to what was shown in Fig. 1A. When Euclidean distance was used, the replicate samples from 6 PDX models were not clustered with each other (Table 1; Additional file 1: Figure S1C, left panel), which indicates that Z-score transformation cannot resolve the normalization issue for this model. We also performed TMM normalization on TPM data. Following this approach, the three replicates for model 475296-252-R did cluster with each other (Additional file 1: Figure S3A). However, the scatter plots of TMM-normalized TPM data for pairwise comparison of all genes among the three replicates still demonstrated a coordinated shift for highly expressed genes (Additional file 1: Figure S3B). Moreover, the median CV of TMM-normalized TPM data (pink bar, Additional file 1: Figure S4) for all genes across the replicates for each model were much higher than those based on TMM-normalized count data (gold bar, Additional file 1: Figure S4).

### A few very highly expressed genes skewed the distribution of TPM expression values

**Fig. 4:**
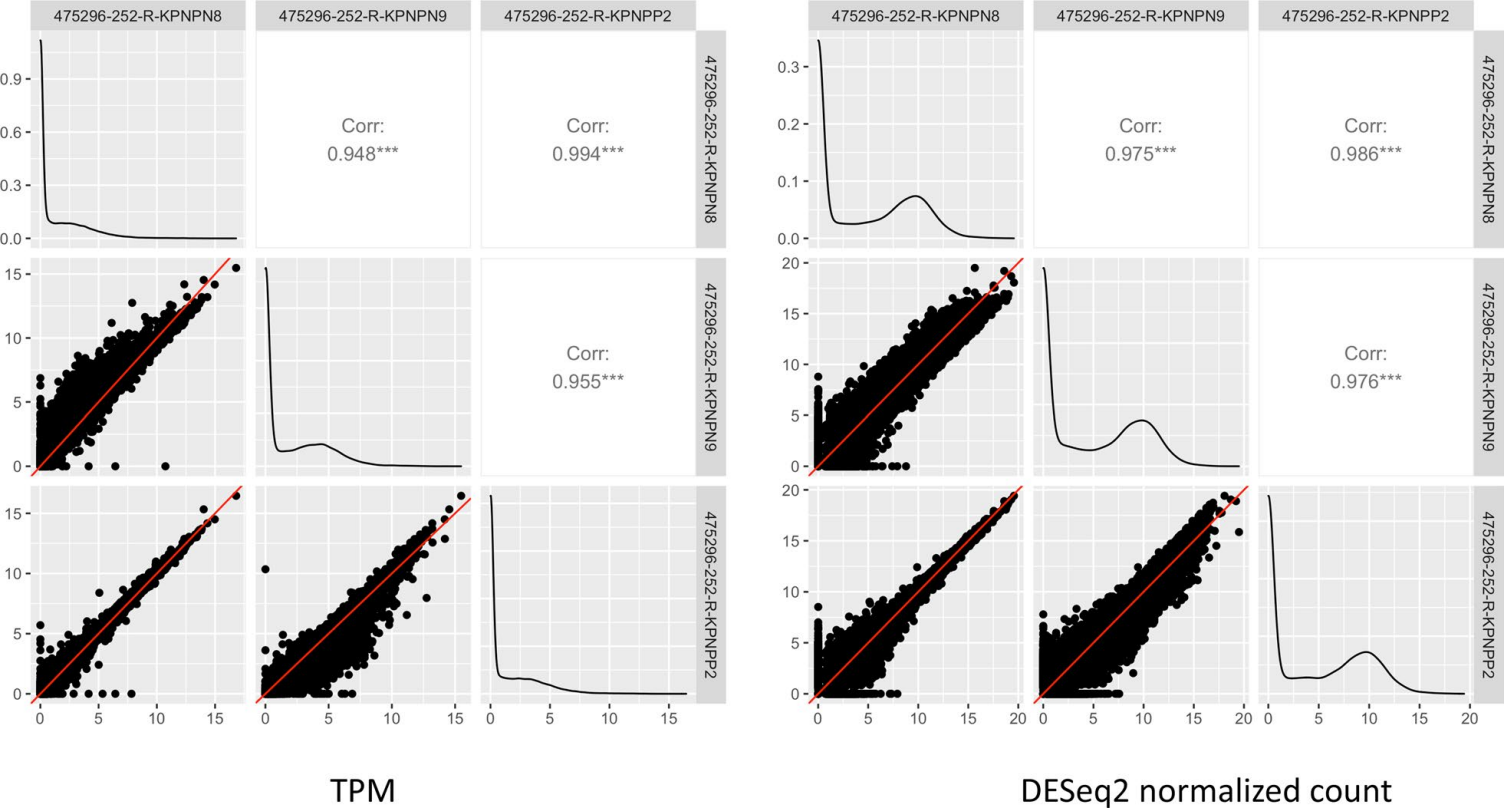
**A** Pairwise scatter plots comparing TPM values for all genes between replicate samples of PDX model 475296-252-R. **B** Pairwise scatter plots comparing DESeq2 normalized count values for all genes between replicate samples of PDX model 475296-252-R. The x- and y- axes are normalized log_2_ counts on all pairwise scatter plots. Plots along the diagonal represent the density of the respective variable

In order to identify factors that possibly contribute to the potentially problematic issue of TPM values across replicate samples, we took a closer look at the pairwise scatter plots for expression of all genes among the 3 replicate samples from PDX model 475296-252-R (samples KPNPP2, KPNPN8, and KPNPN9)—the model for which replicate samples did not cluster with each other in the hierarchical cluster analysis (Fig. 4). Figure 4A contains scatter plots using TPM values, while the scatter plots in Fig. 4B were drawn using DESeq2-normalized count values. In the TPM based scatter plots, there was an upward shift pattern (away from the 45-degree line) between KPNPN8 and KPNPN9, and a downward shift pattern between KPNPP2 and KPNPN9. Those patterns implied that the expression of the majority of genes was systematically skewed towards larger pairwise differences between samples from the same model, which we do not expect to see in replicate samples. When we used the normalized count data, these patterns disappeared, which supports the use of DESeq2 for proper RNA-seq data normalization.

**Fig. 5:**
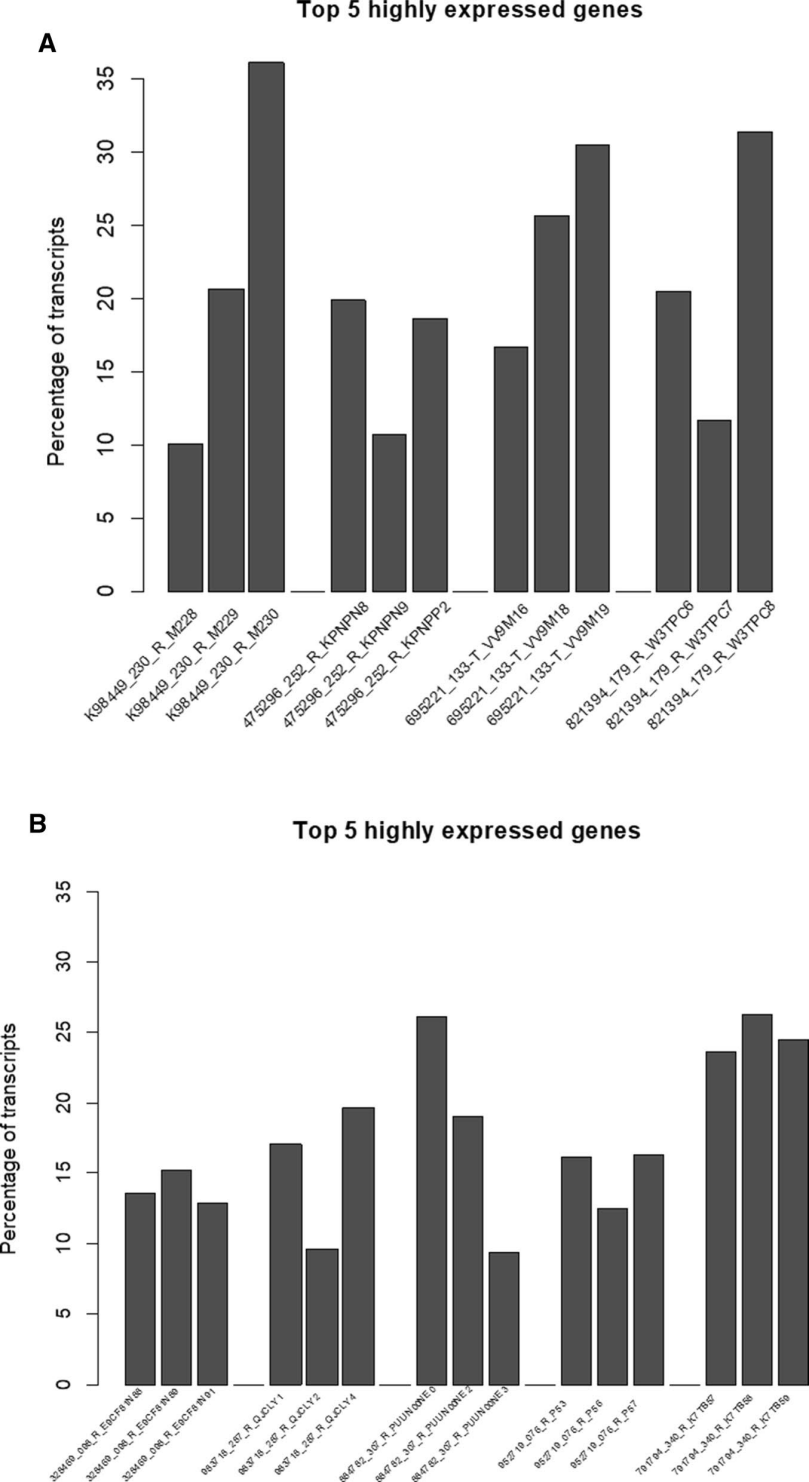
**A** Bar plot of the sum of TPM values for the top 5 most highly expressed genes in four PDX models with the lowest ICC_g_. **B** Bar plot of the sum of TPM values for the top 5 most highly expressed genes in five PDX models with the highest ICC_g_

We extracted the top five most highly expressed genes in the four PDX models for which TPM data had the lowest ICCs compared to the other gene expression measures (models 475296-252-R, 695221-133-T, 821394-179-R, and K98449-230-R, circled in Fig. 3), and calculated the percentage of total TPM assigned to these top five genes in each replicate sample under each model. We found that the proportion of the top five genes differed significantly among replicates for the four models (Fig. 5A). The majority of those genes were either ribosomal RNA or mitochondrial RNAs (Additional file 1: Table S3A). Those four models happened to have the highest median CV values in Fig. 2, and the largest distance in the clustering using TPM data in Fig. 1B and Figure S2. Because the sum of all TPM values is the same for all samples, the fraction of the top five most highly expressed genes in a given sample affects the distribution of the TPM values for the remaining genes in that sample. Therefore, differences in the abundance of the top five most expressed genes are likely to affect the relative abundance of all other transcripts in a sample, thus leading to larger clustering distances, larger median CV values, and lower ICCs.

For comparison, we applied the same procedure to the top five most highly expressed genes in the five PDX models whose TPM data had the lowest median CV values (i.e., models with the least variance between replicates in TPM-quantified gene expression). Among them, while three out of the five models showed minor differences (<  5%) in CVs between the replicates, two of the models still displayed relatively high differences between replicates (Fig. 5B; Additional file 1: Table S3B). We further examined the pairwise scatter plots of the replicate samples for the two models (983718-287-R and 884782-307-R) and found that in both cases, there was only one very highly expressed outlier gene driving the trend (i.e., 5S_rRNA) in each model, while gene expression values for the other genes were very well aligned, as indicated by the distribution of points around the 45-degree line in the pairwise scatter plots of all genes among the replicates (Additional file 1: Figure S8A, B).

## Discussion

Choosing an appropriate gene quantification measure is a key step in the downstream analysis of RNA-seq data. We explored the performance of a few widely used measures on a comprehensive collection of replicate samples of 20 PDX models in RNA-seq experiments across 15 cancer types to address this question. We compared TPM, FPKM, normalized counts using DESeq2 and TMM approaches, and we examined the impact of using variance stabilizing Z-score normalization on TPM-level data as well. We found that for our datasets, both DESeq2 normalized count data (i.e., median of ratios method) and TMM normalized count data generally performed better than the other quantification measures.

Each normalization method comes with a set of assumptions; thus, the validity of downstream analysis results depend on whether the experimental setup is congruent with the assumptions [32]. For instance, library size normalization approaches such as RPKM and its variant FPKM rely on the assumption that the total amount of mRNA/cell is the same for all conditions. In contrast, approaches such as TMM and DESeq perform normalization by comparing read count distribution across samples, and assume symmetrical differential expression between conditions (i.e., most genes are not differentially expressed between two conditions, and the number of upregulated and downregulated genes is comparable) [20, 21, 32]. In these cases, all genes are scaled by the same normalization factor—whether they are differentially expressed or not—derived from the distance to an empirical reference sample. In practice, RPKM/FPKM and TPM tend to perform worse than distribution normalization methods because the requirement for the same amount of mRNA/cell does not hold, as substantiated by multiple reports of a few highly expressed genes dominating the number of mapped reads [9, 33, 34]. We made a similar observation in our study of 61 PDX samples (Fig. 5; Additional file 1: Table S2).

Reproducibility data (i.e., a dataset comprised of n sets of replicate samples) can be used effectively to evaluate the performance of different normalization methods. Wagner et al*.* [35] discussed some of the benefits of TPM over FPKM and advocated for the use of TPM based on a small data set of six human tissue/cell samples with only two replicates. Additionally, Abrams et al*.* [37] recently published a protocol to evaluate RNA sequencing normalization methods using a pool of well-characterized RNA samples from the Universal Human Reference RNA (UHRR, from ten pooled cancer cell lines, Agilent Technologies, Inc.) and the Human Brain Reference RNA (HBRR, from multiple brain regions of 23 donors, Life Technologies, Inc.) [36, 37]. The authors performed a two-way ANOVA to assess the relative contribution of biology and technology to the measured gene expression variability, and concluded that TPM was the best performing normalization method because it retained biological variability without introducing much additional bias in their dataset of reference cancer cell lines and human brain samples [37]. Their conclusion was based on the analysis of technical replicates (i.e., same samples sequenced in different laboratories) from pooled human cancer cell lines and human brain tissue samples. A recent study from The Jackson Laboratory outlined a genomic data analysis workflow for PDX tumor samples from 455 models, wherein gene expression estimates were determined using RSEM. Both expected count and TPM data were used in their data analysis examples. However, recommendations were not made on optimal RNA-seq quantification measures for cross-sample comparison as the study did not include a systematic comparison of replicate samples [38].

The focus of our study was PDX samples, which are inherently more heterogeneous than cell lines, thereby making selection of a sequencing data normalization method critical. We opted to use early passage PDXs because they encountered less evolutionary pressure to adapt to a new environment. Therefore the PDX replicates from 20 models that we chose are more genetically similar to the original tumor [39]. Furthermore, noise may have been introduced in the RNA extraction and library preparation steps; and the presence of host mouse cells within the xenografted tumor requiring a bioinformatic filtration step, constitutes a further challenge [40–42].

Using the data in NCI PDMR database we compared different RNA-seq quantification measures in 20 histologically diverse PDX samples with three or more replicates to evaluate the three different quantification measures TPM, FPKM, and normalized count. In our study, TPM seemed to perform the worst according to multiple evaluation metrics. Similar to FPKM, TPM performed poorly when replicate samples from the same PDX model had heterogeneous transcript distributions, as seen in Fig. 4; that is, highly and differentially expressed features can skew the count distribution. As pointed out by Pachter [43], the dependency of TPM on effective lengths means that abundances reported in TPM are very sensitive to the estimates of effective length. Zhao et al*.* [10] suggested a workflow to follow for analysis of TPM or FPKM/RPKM level-data, which includes different paths depending on whether the same protocol and library were used, and whether the fractions of ribosomal, mitochondrial, and globin RNA were similar. In our examples, the top five most highly expressed genes have imbalanced fractions across the replicates hence leading to larger variations. Additionally, we noted that the genes with the highest TPM expression levels tended to overrepresent ribosomal and mitochondrial genes (Additional file 1: Table S2). These factors, in addition to differences in sequencing depth, may all contribute to the observed variation between replicate samples in our study, thus cementing the need for a robust normalization routine.

There have been discussions on the pitfalls of using TPM for cross-sample comparisons. These pitfalls will lead to some major problems in downstream analyses for RNA-seq data. For example, when correlation of gene expression values with some other continuous variable across experimental subjects is of interest, one must rely on comparability of gene expression measurements to both reduce technical noise that may attenuate correlations and avoid extreme measurements that could produce spurious correlations. Certain features of the underlying data may adversely affect the performance of some of these quantification methods. For example, high expression of ribosomal RNA may lead to a skewed distribution of TPM-normalized expression measures for a particular sample. Consequently, a computed correlation will not be accurate even if the rank statistics are used because the comparison is at the gene-level. Secondly, for differential expression (DE) analysis, statistical models usually assume that the data follow some probability distribution. Currently, the majority of the DE analysis tools for RNA-seq assume a Poisson/negative binomial distribution for the data. Since TPM/FPKM are not count data, they cannot be modeled using these types of discrete probability distributions. In addition, shrinkage methods implemented in many DE analysis tools require those distribution assumptions to hold, which clearly they will not, for length-normalized measures such as TPM or FPKM/RPKM. Thirdly, some gene set enrichment analysis methods rely on parametric assumptions about the data distribution for calculation of test statistics and p values [e.g. Fisher (LS) statistics]. TPM and FPKM/RPKM may be acceptable to use if the ranks of genes in each sample are used, as opposed to their quantitative expression values. For example, The Broad Institute’s gene set enrichment analysis (GSEA) tool allows users to perform pathway analyses by uploading single rank-based gene list [44, 45]. Finally, our analyses demonstrated that neither Z-score nor additional normalization steps can resolve the potentially problematic issue in TPM data. We recommend using raw count matrix normalized by either DESeq2 or TMM for PDX studies.

As described above, each normalization method is based on its own assumptions. When the assumptions are violated, the method could fail [32]. In this paper, we showed examples of such scenarios where TPM and FPKM did not perform as reliably as normalized counts by DESeq2 or TMM in at least four PDX models. Therefore, it is important to consider context when selecting normalization methods and not arbitrarily use a single method for all purposes [38]. Researchers need to be aware of assumptions made by various methods, and data characteristics that might violate those assumptions, in order to choose the right normalization method for their study.

## Conclusion

Our results strongly support the notion that normalized count data are the preferred quantification measure for between-sample analysis of RNA-seq data generated from tumors grown in PDX models. These quantifications exhibit greater comparability among replicate samples and are more robust to technical artifacts; hence, they should be the first choice whenever cross-sample comparisons are of interest. Further data transformations or normalizations on TPM-level data are not able to resolve potential issues inherent in TPM quantifications. We hope that our findings will promote the use of normalized count data instead of TPM or FPKM/RPKM in PDX studies using RNA-seq to avoid inaccurate results arising from sub-optimal gene expression quantification.

## Supplementary Information

**Additional file 1: Table S1.** Details of the patient-derived xenograft samples used in this study (downloaded on Sept. 16, 2020 from the NCI PDMR database). **Table S2.** Summary statistics for CV values including interquartile range for different quantitative measures. **Table S3A.** Percentage of transcripts representing each of the top five most abundant genes in four PDX models whose TPM data had the highest median CV values. **Table S3B.** Percentage of transcripts representing each of the top five most abundant genes in five PDX models whose TPM data had the lowest median CV values. **Figure S1.** (**A**) Hierarchical clustering of 61 PDX samples using TMM normalized gene-level count data. (**B**) Hierarchical clustering of 61 PDX samples using FPKM data. (**C**) Hierarchical clustering of 61 PDX samples using Z-score on TPM-level data. Distance metric 1-Pearson correlation was used to generate the dendrogram in each right panel and Euclidean distance was used for the dendrogram in each left panel. Discordant models are highlighted with different color labels. **Figure S2.** Maximum distance (1-Pearson correlation) between replicate samples for the four PDX models with high median CV values using different gene expression quantification measures. **Figure S3.** (**A**) Hierarchical clustering of 61 PDX samples using TMM normalized TPM data. Distance metric 1-Pearson correlation was used to generate the dendrogram in the right panel and Euclidean distance was used for the dendrogram in the left panel. (**B**) Pairwise scatter plots comparing normalized TPM values for all genes among replicates of PDX model 475296-252-R. The x- and y- axes are normalized log_2_ counts on all pairwise scatter plots. Plots along the diagonal represent the density of the respective variable. **Figure S4.** Bar plot of median CVs for gene expression levels from replicate samples of each PDX model using different quantification measures. **Figure S5.** (**A**) Pairwise scatter plots comparing TPM values for all genes between replicate samples of PDX model 695221-133-T. (**B**) Pairwise scatter plots comparing DESeq2 normalized count values for all genes between replicate samples of PDX model 695221-133-T. The x- and y-axes are normalized log_2_ counts on all pairwise scatter plots. Plots along the diagonal represent the density of the respective variable. **Figure S6.** (**A**) Pairwise scatter plots comparing TPM values for all genes between replicate samples of PDX model 821394-179-R. (**B**) Pairwise scatter plots comparing DESeq2 normalized count values for all genes between replicate samples of PDX model 821394-179-R. The x- and y-axes are normalized log_2_ counts on all pairwise scatter plots. Plots along the diagonal represent the density of the respective variable. **Figure S7.** (**A**) Pairwise scatter plots comparing TPM values for all genes between replicate samples of PDX model K98449-230-R. (**B**) Pairwise scatter plots comparing DESeq2 normalized count values for all genes between replicate samples of PDX model K98449-230-R. The x- and y-axes are normalized log_2_ counts on all pairwise scatter plots. Plots along the diagonal represent the density of the respective variable. **Figure S8.** (**A**) Pairwise scatter plots comparing TPM values for all genes between replicate samples of PDX model 983718-287-R. (**B**) Pairwise scatter plots comparing TPM values for all genes between replicate samples of PDX model 884782-307-R. The x- and y-axes are normalized log_2_ counts on all pairwise scatter plots. Plots along the diagonal represent the density of the respective variable.

## Data Availability

Data used in this study can be downloaded from https://pdmr.cancer.gov/.

## Abbreviations

TPM: Transcript per Million
RPKM: Reads per kilobase of transcript per Million reads mapped
FPKM: Fragments Per kilobase of transcript per Million reads mapped
PDX: Patient-derived xenograft
PDM: Patient-derived model
PDMR: Patient-derived xenograft repository
TMM: Trimmed mean of M values
CV: Coefficient of variation
ICC: Intraclass correlation coefficient
DE: Differential expression
GSEA: Gene set enrichment analysis
