# Supplementary material for: TPM, FPKM, or Normalized Counts? A Comparative Study of Quantification Measures for the Analysis of RNA-seq Data from the NCI Patient-Derived Models Repository
Source: J Transl Med. 2021 Jun 22;19:269. doi: 10.1186/s12967-021-02936-w (PMC8220791; doi:10.1186/s12967-021-02936-w)
Supplement: Supplementary file 1 — Additional file 1: Table S1. Details of the patient-derived xenograft samples used in this study (downloaded on Sept. 16, 2020 from the NCI PDMR database). Table S2. Summary statistics for CV values including interquartile range for different quantitative measures. Table S3A. Percentage of transcripts representing each of the top five most abundant genes in four PDX models whose TPM data had the highest median CV values. Table S3B. Percentage of transcripts representing each of the top five most abundant genes in five PDX models whose TPM data had the lowest median CV values. Figure S1. (A) Hierarchical clustering of 61 PDX samples using TMM normalized gene-level count data. (B) Hierarchical clustering of 61 PDX samples using FPKM data. (C) Hierarchical clustering of 61 PDX samples using Z-score on TPM-level data. Distance metric 1-Pearson correlation was used to generate the dendrogram in each right panel and Euclidean distance was used for the dendrogram in each left panel. Discordant models are highlighted with different color labels. Figure S2. Maximum distance (1-Pearson correlation) between replicate samples for the four PDX models with high median CV values using different gene expression quantification measures. Figure S3. (A) Hierarchical clustering of 61 PDX samples using TMM normalized TPM data. Distance metric 1-Pearson correlation was used to generate the dendrogram in the right panel and Euclidean distance was used for the dendrogram in the left panel. (B) Pairwise scatter plots comparing normalized TPM values for all genes among replicates of PDX model 475296-252-R. The x- and y- axes are normalized log2 counts on all pairwise scatter plots. Plots along the diagonal represent the density of the respective variable. Figure S4. Bar plot of median CVs for gene expression levels from replicate samples of each PDX model using different quantification measures. Figure S5. (A) Pairwise scatter plots comparing TPM values for all genes between replicate sample [file 12967_2021_2936_MOESM1_ESM.pdf]

Figure S1A

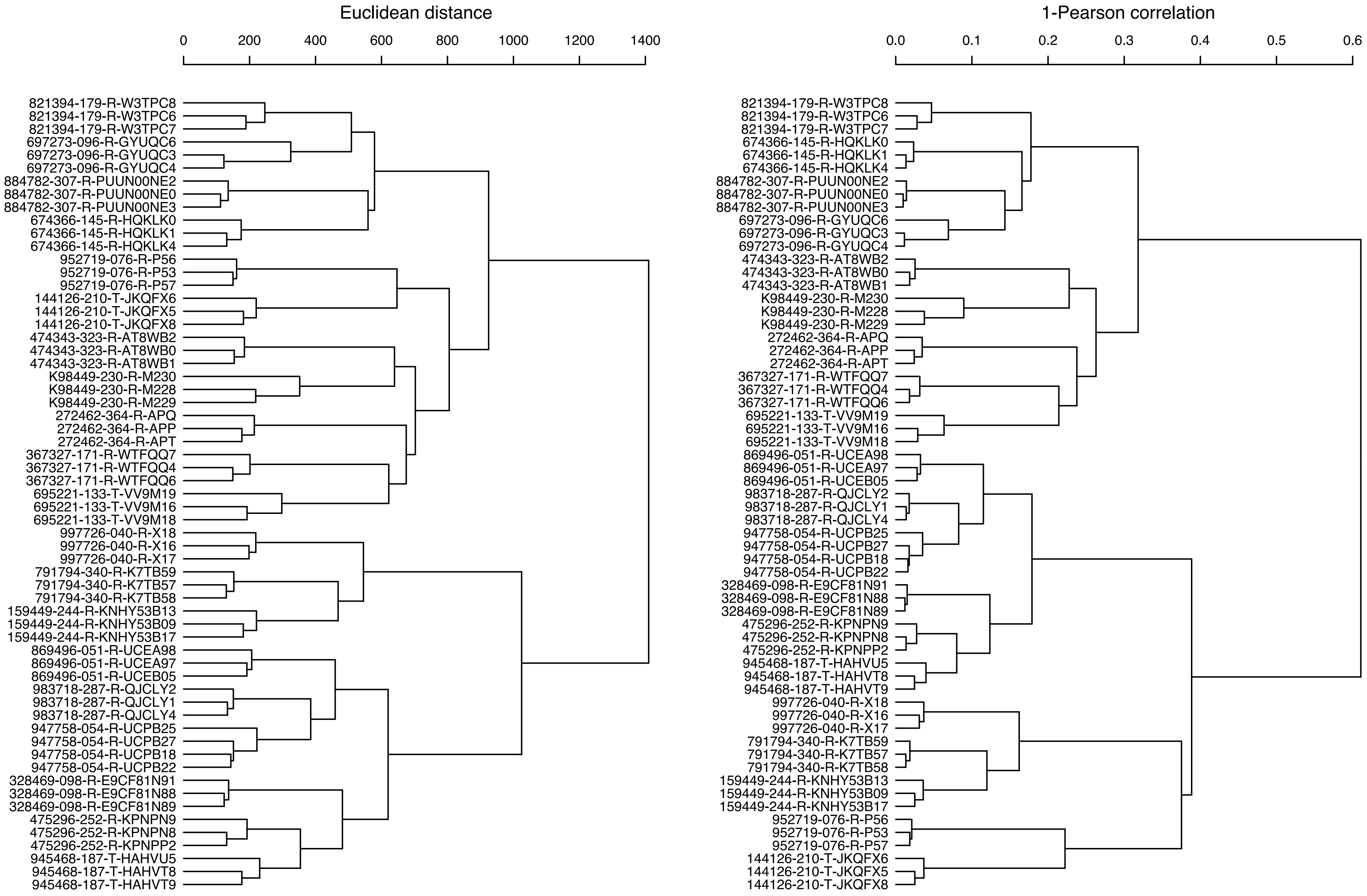
Euclidean distance
0
200
400
600
800
1000
1200
1400
1-Pearson correlation
0.0
0.1
0.2
0.3
0.4
0.5
0.6
821394-179-R-W3TPC8
821394-179-R-W3TPC6
821394-179-R-W3TPC7
697273-096-R-GYUQC6
697273-096-R-GYUQC3
697273-096-R-GYUQC4
884782-307-R-PUUN00NE2
884782-307-R-PUUN00NE0
884782-307-R-PUUN00NE3
674366-145-R-HQKLK0
674366-145-R-HQKLK1
674366-145-R-HQKLK4
952719-076-R-P56
952719-076-R-P53
952719-076-R-P57
144126-210-T-JKQFX6
144126-210-T-JKQFX5
144126-210-T-JKQFX8
474343-323-R-AT8WB2
474343-323-R-AT8WB0
474343-323-R-AT8WB1
K98449-230-R-M230
K98449-230-R-M228
K98449-230-R-M229
272462-364-R-APQ
272462-364-R-APP
272462-364-R-APT
367327-171-R-WTFQQ7
367327-171-R-WTFQQ4
367327-171-R-WTFQQ6
695221-133-T-VV9M19
695221-133-T-VV9M16
695221-133-T-VV9M18
997726-040-R-X18
997726-040-R-X16
997726-040-R-X17
791794-340-R-K7TB59
791794-340-R-K7TB57
791794-340-R-K7TB58
159449-244-R-KNHY53B13
159449-244-R-KNHY53B09
159449-244-R-KNHY53B17
869496-051-R-UCEA98
869496-051-R-UCEA97
869496-051-R-UCEB05
983718-287-R-QJCLY2
983718-287-R-QJCLY1
983718-287-R-QJCLY4
947758-054-R-UCPB25
947758-054-R-UCPB27
947758-054-R-UCPB18
947758-054-R-UCPB22
328469-098-R-E9CF81N91
328469-098-R-E9CF81N88
328469-098-R-E9CF81N89
475296-252-R-KPNPN9
475296-252-R-KPNPN8
475296-252-R-KPNPP2
945468-187-T-HAHVU5
945468-187-T-HAHVT8
945468-187-T-HAHVT9

Figure S1B

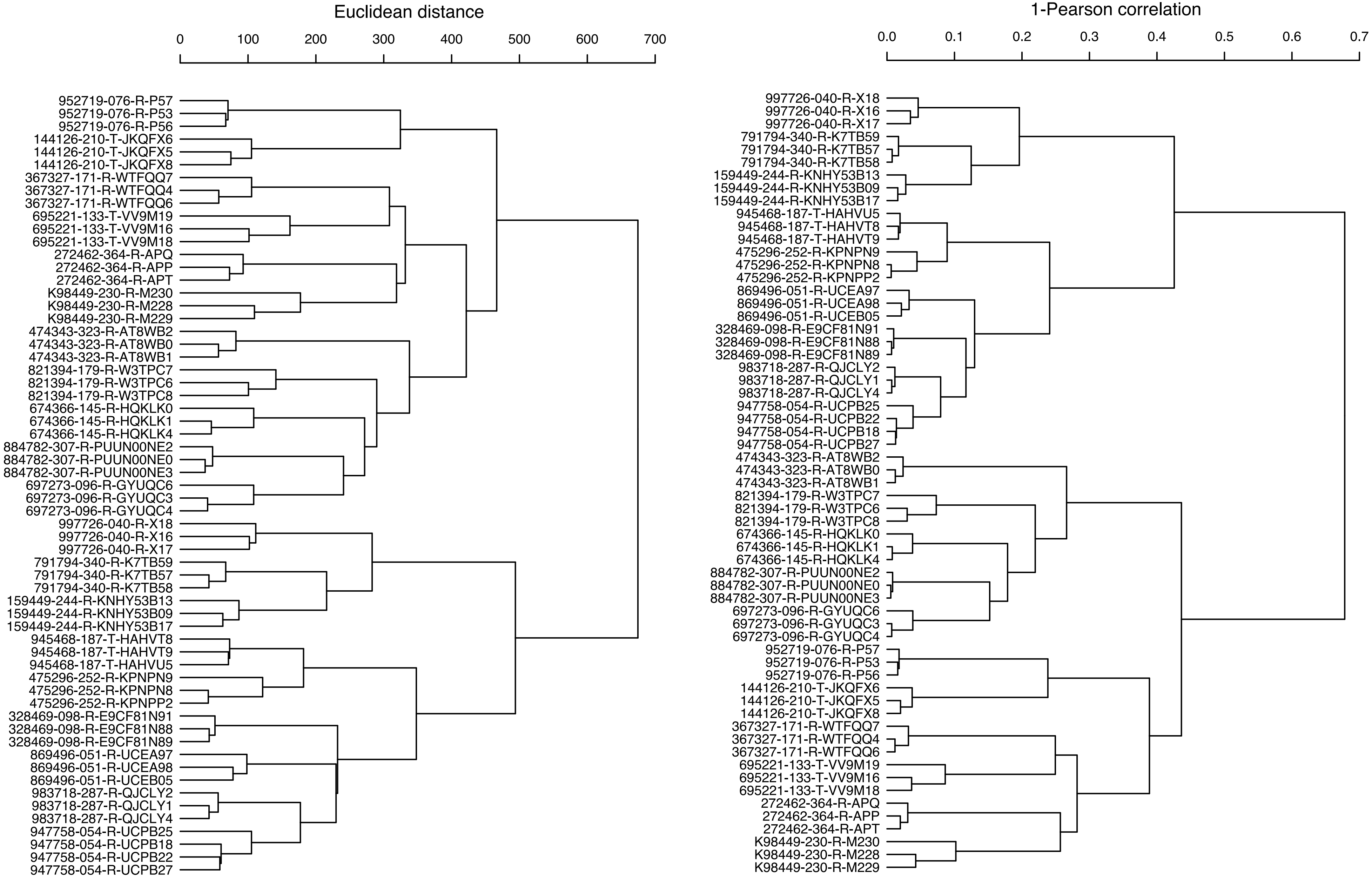

Euclidean distance
0
100
200
300
400
500
600
700
952719-076-R-P57
952719-076-R-P53
952719-076-R-P56
144126-210-T-JKQFX6
144126-210-T-JKQFX5
144126-210-T-JKQFX8
367327-171-R-WTFQQ7
367327-171-R-WTFQQ4
367327-171-R-WTFQQ6
695221-133-T-VV9M19
695221-133-T-VV9M16
695221-133-T-VV9M18
272462-364-R-APQ
272462-364-R-APP
272462-364-R-APT
K98449-230-R-M230
K98449-230-R-M228
K98449-230-R-M229
474343-323-R-AT8WB2
474343-323-R-AT8WB0
474343-323-R-AT8WB1
821394-179-R-W3TPC7
821394-179-R-W3TPC6
821394-179-R-W3TPC8
674366-145-R-HQKLK0
674366-145-R-HQKLK1
674366-145-R-HQKLK4
884782-307-R-PUUN00NE2
884782-307-R-PUUN00NE0
884782-307-R-PUUN00NE3
697273-096-R-GYUQC6
697273-096-R-GYUQC3
697273-096-R-GYUQC4
997726-040-R-X18
997726-040-R-X16
997726-040-R-X17
791794-340-R-K7TB59
791794-340-R-K7TB57
791794-340-R-K7TB58
159449-244-R-KNHY53B13
159449-244-R-KNHY53B09
159449-244-R-KNHY53B17
945468-187-T-HAHVT8
945468-187-T-HAHVT9
945468-187-T-HAHVU5
475296-252-R-KPNPN9
475296-252-R-KPNPN8
475296-252-R-KPNPP2
328469-098-R-E9CF81N91
328469-098-R-E9CF81N88
328469-098-R-E9CF81N89
869496-051-R-UCEA97
869496-051-R-UCEA98
869496-051-R-UCEB05
983718-287-R-QJCLY2
983718-287-R-QJCLY1
983718-287-R-QJCLY4
947758-054-R-UCPB25
947758-054-R-UCPB18
947758-054-R-UCPB22
947758-054-R-UCPB27
1-Pearson correlation
0.0
0.1
0.2
0.3
0.4
0.5
0.6
0.7
997726-040-R-X18
997726-040-R-X16
997726-040-R-X17
791794-340-R-K7TB59
791794-340-R-K7TB57
791794-340-R-K7TB58
159449-244-R-KNHY53B13
159449-244-R-KNHY53B09
159449-244-R-KNHY53B17
945468-187-T-HAHVU5
945468-187-T-HAHVT8
945468-187-T-HAHVT9
475296-252-R-KPNPN9
475296-252-R-KPNPN8
475296-252-R-KPNPP2
869496-051-R-UCEA97
869496-051-R-UCEA98
869496-051-R-UCEB05
328469-098-R-E9CF81N91
328469-098-R-E9CF81N88
328469-098-R-E9CF81N89
983718-287-R-QJCLY2
983718-287-R-QJCLY1
983718-287-R-QJCLY4
947758-054-R-UCPB25
947758-054-R-UCPB22
947758-054-R-UCPB18
947758-054-R-UCPB27
474343-323-R-AT8WB2
474343-323-R-AT8WB0
474343-323-R-AT8WB1
821394-179-R-W3TPC7
821394-179-R-W3TPC6
821394-179-R-W3TPC8
674366-145-R-HQKLK0
674366-145-R-HQKLK1
674366-145-R-HQKLK4
884782-307-R-PUUN00NE2
884782-307-R-PUUN00NE0
884782-307-R-PUUN00NE3
697273-096-R-GYUQC6
697273-096-R-GYUQC3
697273-096-R-GYUQC4
952719-076-R-P57
952719-076-R-P53
952719-076-R-P56
144126-210-T-JKQFX6
144126-210-T-JKQFX5
144126-210-T-JKQFX8
367327-171-R-WTFQQ7
367327-171-R-WTFQQ4
367327-171-R-WTFQQ6
695221-133-T-VV9M19
695221-133-T-VV9M16
695221-133-T-VV9M18
272462-364-R-APQ
272462-364-R-APP
272462-364-R-APT
K98449-230-R-M230
K98449-230-R-M228
K98449-230-R-M229

Figure S1C

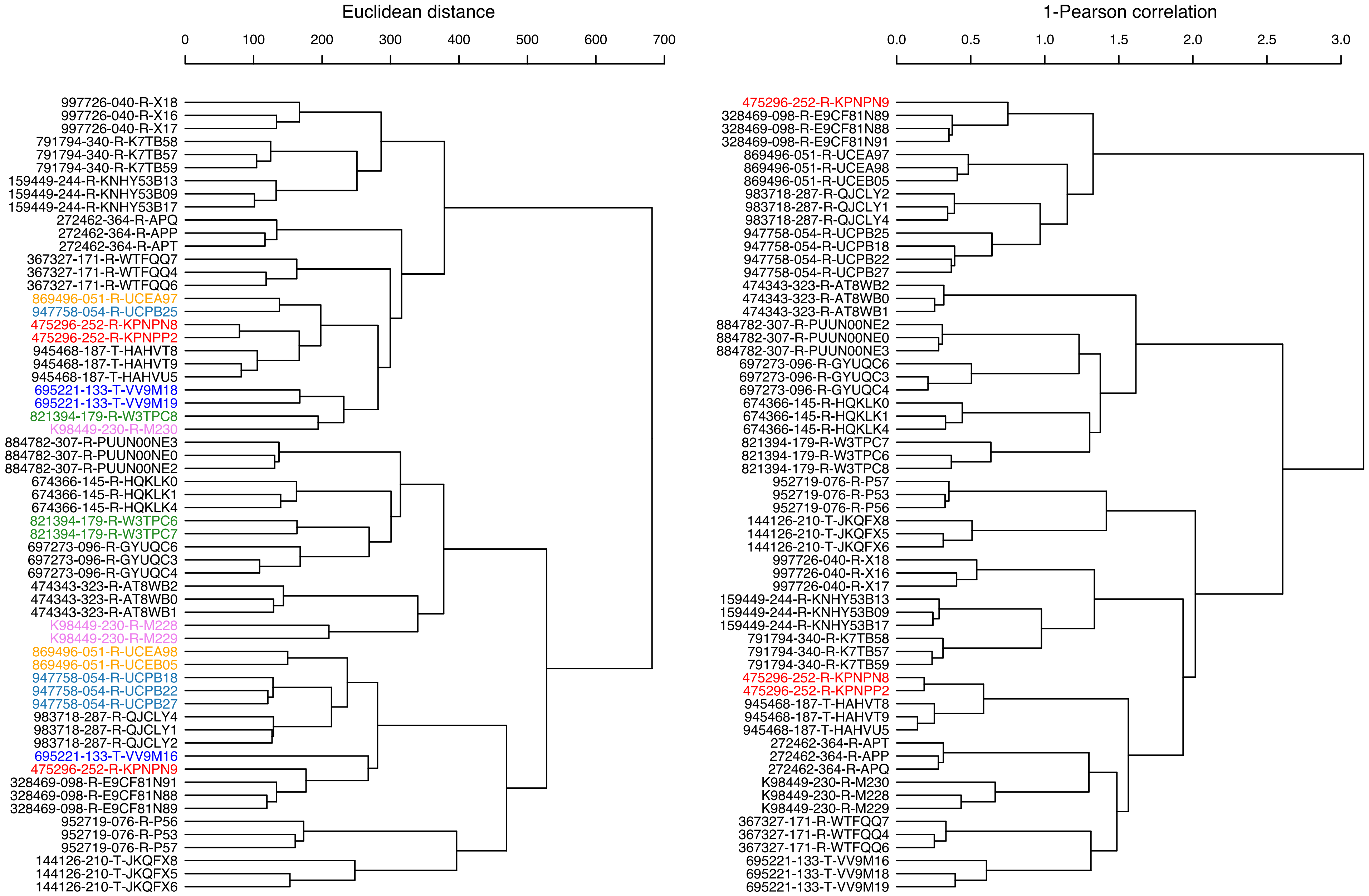

Euclidean distance
0
100
200
300
400
500
600
700
997726-040-R-X18
997726-040-R-X16
997726-040-R-X17
791794-340-R-K7TB58
791794-340-R-K7TB57
791794-340-R-K7TB59
159449-244-R-KNHY53B13
159449-244-R-KNHY53B09
159449-244-R-KNHY53B17
272462-364-R-APQ
272462-364-R-APP
272462-364-R-APT
367327-171-R-WTFQQ7
367327-171-R-WTFQQ4
367327-171-R-WTFQQ6
869496-051-R-UCEA97
947758-054-R-UCPB25
475296-252-R-KPNPN8
475296-252-R-KPNPP2
945468-187-T-HAHVT8
945468-187-T-HAHVT9
945468-187-T-HAHVU5
695221-133-T-VV9M18
695221-133-T-VV9M19
821394-179-R-W3TPC8
K98449-230-R-M230
884782-307-R-PUUN00NE3
884782-307-R-PUUN00NE0
884782-307-R-PUUN00NE2
674366-145-R-HQKLK0
674366-145-R-HQKLK1
674366-145-R-HQKLK4
821394-179-R-W3TPC6
821394-179-R-W3TPC7
697273-096-R-GYUQC6
697273-096-R-GYUQC3
697273-096-R-GYUQC4
474343-323-R-AT8WB2
474343-323-R-AT8WB0
474343-323-R-AT8WB1
K98449-230-R-M228
K98449-230-R-M229
869496-051-R-UCEA98
869496-051-R-UCEB05
947758-054-R-UCPB18
947758-054-R-UCPB22
947758-054-R-UCPB27
983718-287-R-QJCLY4
983718-287-R-QJCLY1
983718-287-R-QJCLY2
695221-133-T-VV9M16
475296-252-R-KPNPN9
328469-098-R-E9CF81N91
328469-098-R-E9CF81N88
328469-098-R-E9CF81N89
952719-076-R-P56
952719-076-R-P53
952719-076-R-P57
144126-210-T-JKQFX8
144126-210-T-JKQFX5
144126-210-T-JKQFX6
1-Pearson correlation
0.0
0.5
1.0
1.5
2.0
2.5
3.0
475296-252-R-KPNPN9
328469-098-R-E9CF81N89
328469-098-R-E9CF81N88
328469-098-R-E9CF81N91
869496-051-R-UCEA97
869496-051-R-UCEA98
869496-051-R-UCEB05
983718-287-R-QJCLY2
983718-287-R-QJCLY1
983718-287-R-QJCLY4
947758-054-R-UCPB25
947758-054-R-UCPB18
947758-054-R-UCPB22
947758-054-R-UCPB27
474343-323-R-AT8WB2
474343-323-R-AT8WB0
474343-323-R-AT8WB1
884782-307-R-PUUN00NE2
884782-307-R-PUUN00NE0
884782-307-R-PUUN00NE3
697273-096-R-GYUQC6
697273-096-R-GYUQC3
697273-096-R-GYUQC4
674366-145-R-HQKLK0
674366-145-R-HQKLK1
674366-145-R-HQKLK4
821394-179-R-W3TPC7
821394-179-R-W3TPC6
821394-179-R-W3TPC8
952719-076-R-P57
952719-076-R-P53
952719-076-R-P56
144126-210-T-JKQFX8
144126-210-T-JKQFX5
144126-210-T-JKQFX6
997726-040-R-X18
997726-040-R-X16
997726-040-R-X17
159449-244-R-KNHY53B13
159449-244-R-KNHY53B09
159449-244-R-KNHY53B17
791794-340-R-K7TB58
791794-340-R-K7TB57
791794-340-R-K7TB59
475296-252-R-KPNPN8
475296-252-R-KPNPP2
945468-187-T-HAHVT8
945468-187-T-HAHVT9
945468-187-T-HAHVU5
272462-364-R-APT
272462-364-R-APP
272462-364-R-APQ
K98449-230-R-M230
K98449-230-R-M228
K98449-230-R-M229
367327-171-R-WTFQQ7
367327-171-R-WTFQQ4
367327-171-R-WTFQQ6
695221-133-T-VV9M16
695221-133-T-VV9M18
695221-133-T-VV9M19

Figure S2

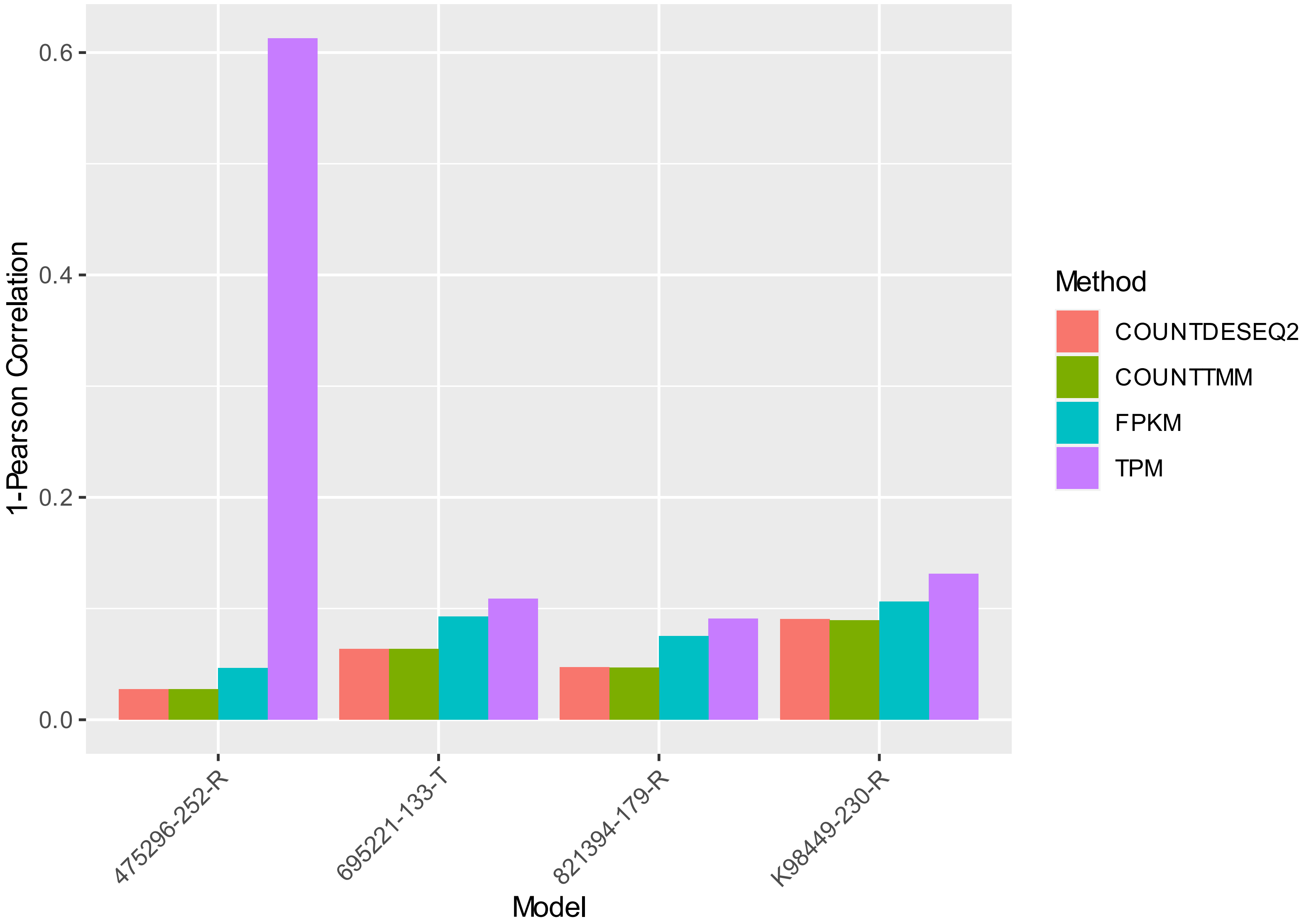

1-Pearson Correlation
0.6
0.4
0.2
0.0
475296-252-R
695221-133-T
821394-179-R
K98449-230-R
Model
Method
COUNTDESEQ2
COUNTTMM
FPKM
TPM

Figure S3A

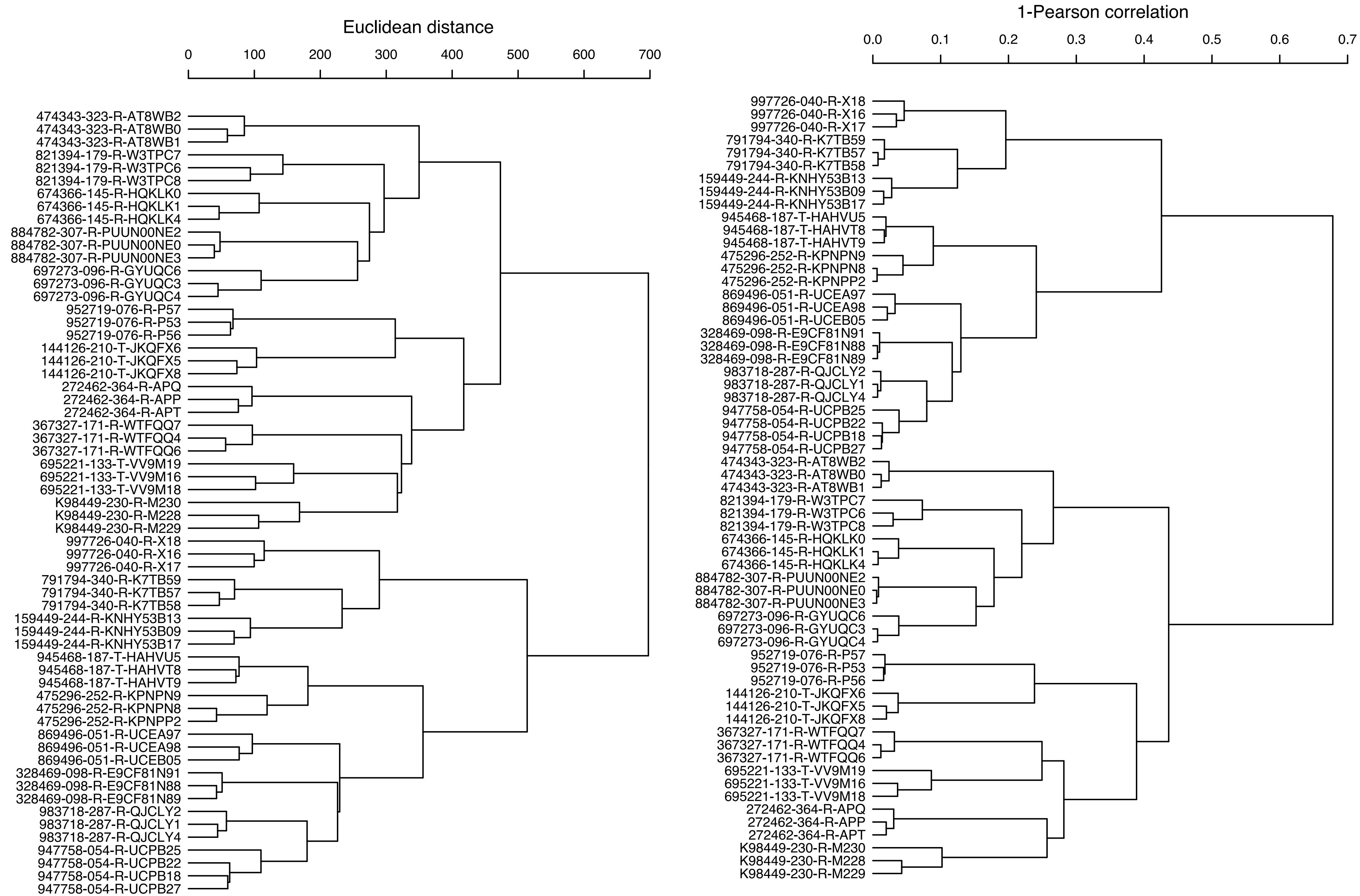

Euclidean distance
0
100
200
300
400
500
600
700
474343-323-R-AT8WB2
474343-323-R-AT8WB0
474343-323-R-AT8WB1
821394-179-R-W3TPC7
821394-179-R-W3TPC6
821394-179-R-W3TPC8
674366-145-R-HQKLK0
674366-145-R-HQKLK1
674366-145-R-HQKLK4
884782-307-R-PUUN00NE2
884782-307-R-PUUN00NE0
884782-307-R-PUUN00NE3
697273-096-R-GYUQC6
697273-096-R-GYUQC3
697273-096-R-GYUQC4
952719-076-R-P57
952719-076-R-P53
952719-076-R-P56
144126-210-T-JKQFX6
144126-210-T-JKQFX5
144126-210-T-JKQFX8
272462-364-R-APQ
272462-364-R-APP
272462-364-R-APT
367327-171-R-WTFQQ7
367327-171-R-WTFQQ4
367327-171-R-WTFQQ6
695221-133-T-VV9M19
695221-133-T-VV9M16
695221-133-T-VV9M18
K98449-230-R-M230
K98449-230-R-M228
K98449-230-R-M229
997726-040-R-X18
997726-040-R-X16
997726-040-R-X17
791794-340-R-K7TB59
791794-340-R-K7TB57
791794-340-R-K7TB58
159449-244-R-KNHY53B13
159449-244-R-KNHY53B09
159449-244-R-KNHY53B17
945468-187-T-HAHVU5
945468-187-T-HAHVT8
945468-187-T-HAHVT9
475296-252-R-KPNPN9
475296-252-R-KPNPN8
475296-252-R-KPNPP2
869496-051-R-UCEA97
869496-051-R-UCEA98
869496-051-R-UCEB05
328469-098-R-E9CF81N91
328469-098-R-E9CF81N88
328469-098-R-E9CF81N89
983718-287-R-QJCLY2
983718-287-R-QJCLY1
983718-287-R-QJCLY4
947758-054-R-UCPB25
947758-054-R-UCPB22
947758-054-R-UCPB18
947758-054-R-UCPB27
1-Pearson correlation
0.0
0.1
0.2
0.3
0.4
0.5
0.6
0.7
997726-040-R-X18
997726-040-R-X16
997726-040-R-X17
791794-340-R-K7TB59
791794-340-R-K7TB57
791794-340-R-K7TB58
159449-244-R-KNHY53B13
159449-244-R-KNHY53B09
159449-244-R-KNHY53B17
945468-187-T-HAHVU5
945468-187-T-HAHVT8
945468-187-T-HAHVT9
475296-252-R-KPNPN9
475296-252-R-KPNPN8
475296-252-R-KPNPP2
869496-051-R-UCEA97
869496-051-R-UCEA98
869496-051-R-UCEB05
328469-098-R-E9CF81N91
328469-098-R-E9CF81N88
328469-098-R-E9CF81N89
983718-287-R-QJCLY2
983718-287-R-QJCLY1
983718-287-R-QJCLY4
947758-054-R-UCPB25
947758-054-R-UCPB22
947758-054-R-UCPB18
947758-054-R-UCPB27
474343-323-R-AT8WB2
474343-323-R-AT8WB0
474343-323-R-AT8WB1
821394-179-R-W3TPC7
821394-179-R-W3TPC6
821394-179-R-W3TPC8
674366-145-R-HQKLK0
674366-145-R-HQKLK1
674366-145-R-HQKLK4
884782-307-R-PUUN00NE2
884782-307-R-PUUN00NE0
884782-307-R-PUUN00NE3
697273-096-R-GYUQC6
697273-096-R-GYUQC3
697273-096-R-GYUQC4
952719-076-R-P57
952719-076-R-P53
952719-076-R-P56
144126-210-T-JKQFX6
144126-210-T-JKQFX5
144126-210-T-JKQFX8
367327-171-R-WTFQQ7
367327-171-R-WTFQQ4
367327-171-R-WTFQQ6
695221-133-T-VV9M19
695221-133-T-VV9M16
695221-133-T-VV9M18
272462-364-R-APQ
272462-364-R-APP
272462-364-R-APT
K98449-230-R-M230
K98449-230-R-M228
K98449-230-R-M229

Figure S3B

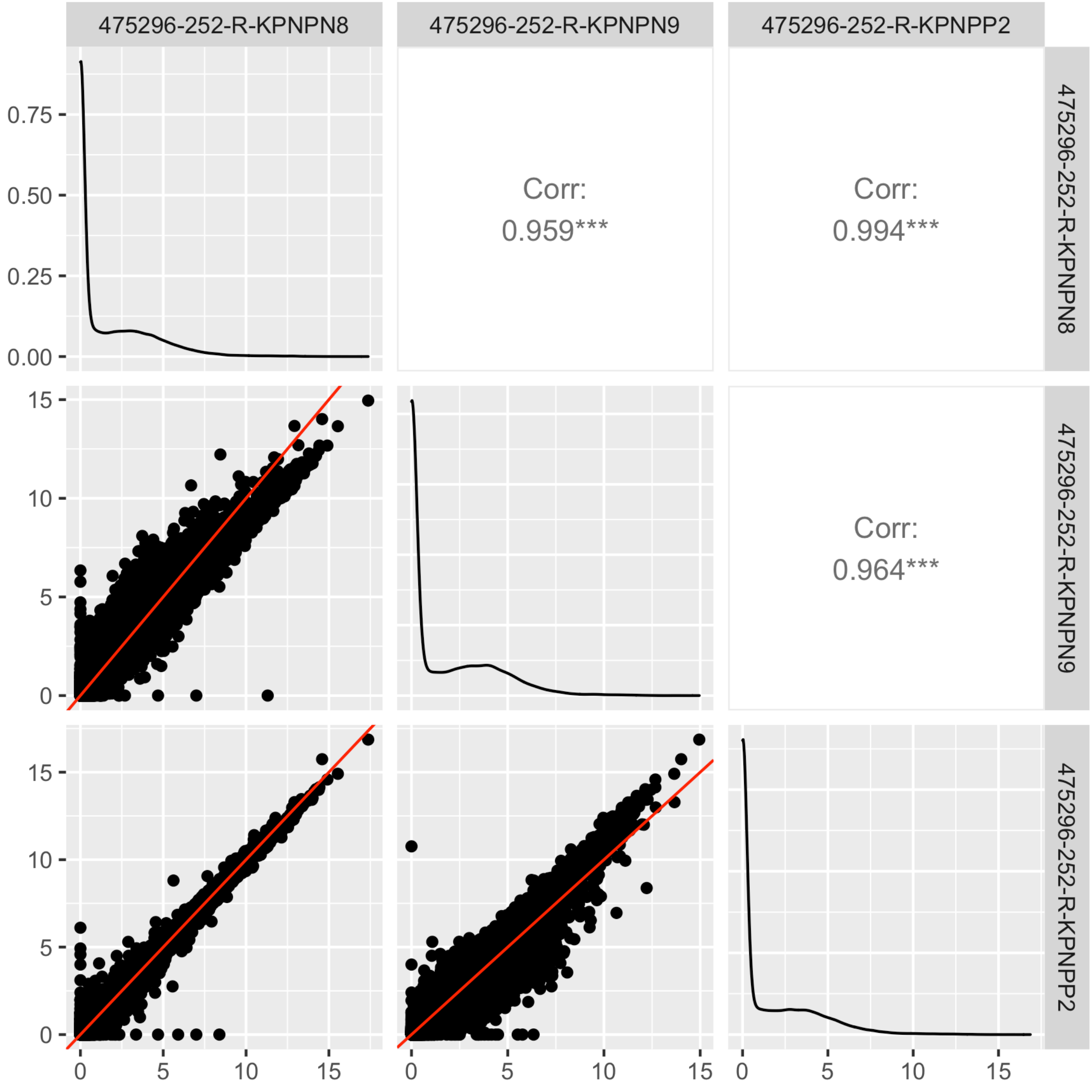

475296-252-R-KPNPN8
475296-252-R-KPNPN9
475296-252-R-KPNPP2
Corr:
0.959***
Corr:
0.994***
Corr:
0.964***
0.75
0.50
0.25
0.00
15
10
5
0

Figure S4

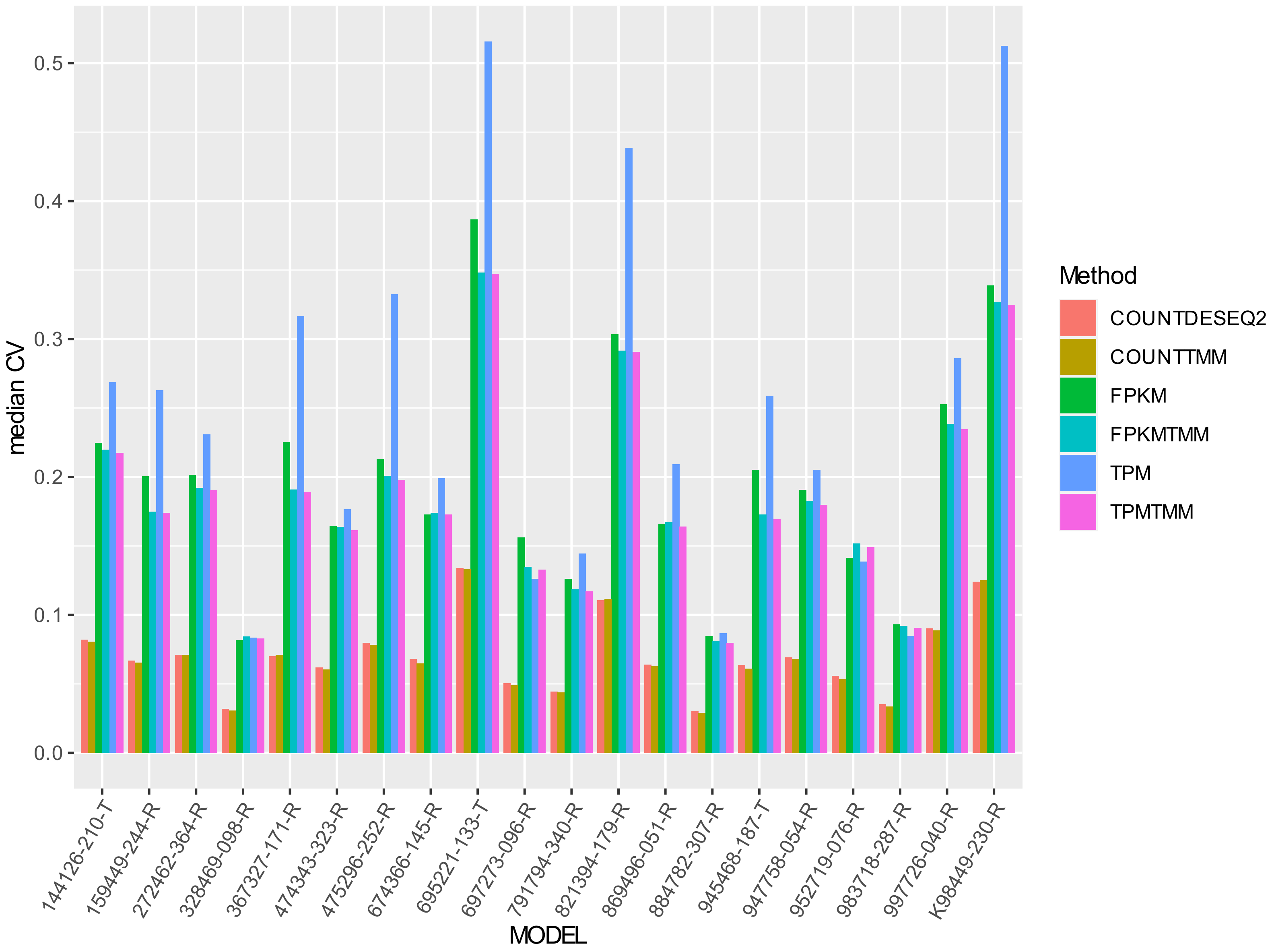

median CV
0.0
0.1
0.2
0.3
0.4
0.5
144126-210-T
159449-244-R
272462-364-R
328469-098-R
367327-171-R
474343-323-R
475296-252-R
674366-145-R
695221-133-T
697273-096-R
791794-340-R
821394-179-R
869496-051-R
884782-307-R
945468-187-T
947758-054-R
952719-076-R
983718-287-R
997726-040-R
K98449-230-R
MODEL
Method
COUNTDESEQ2
COUNTTMM
FPKM
FPKMTMM
TPM
TPMTMM

Figure S5A

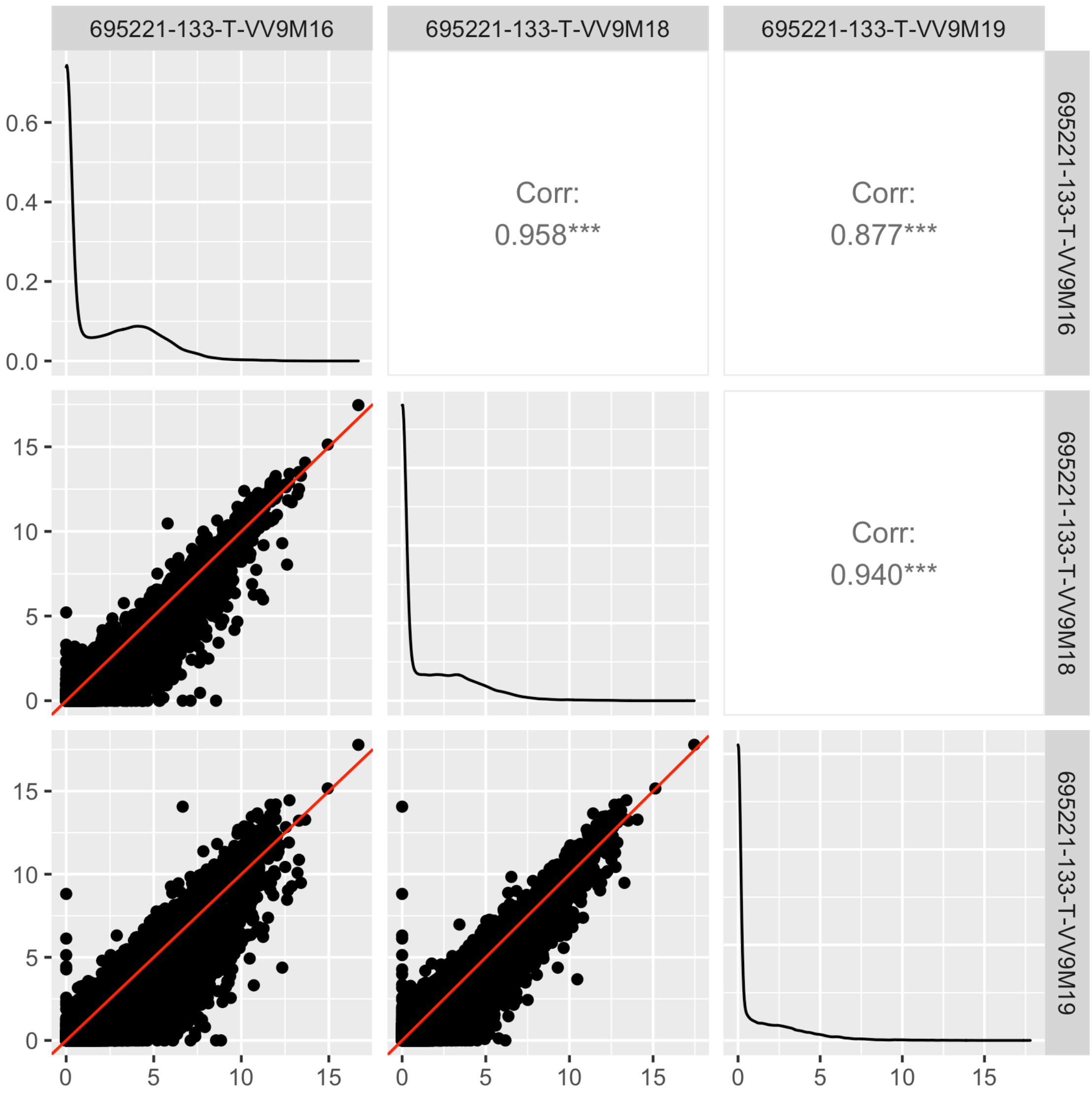

Figure S5B

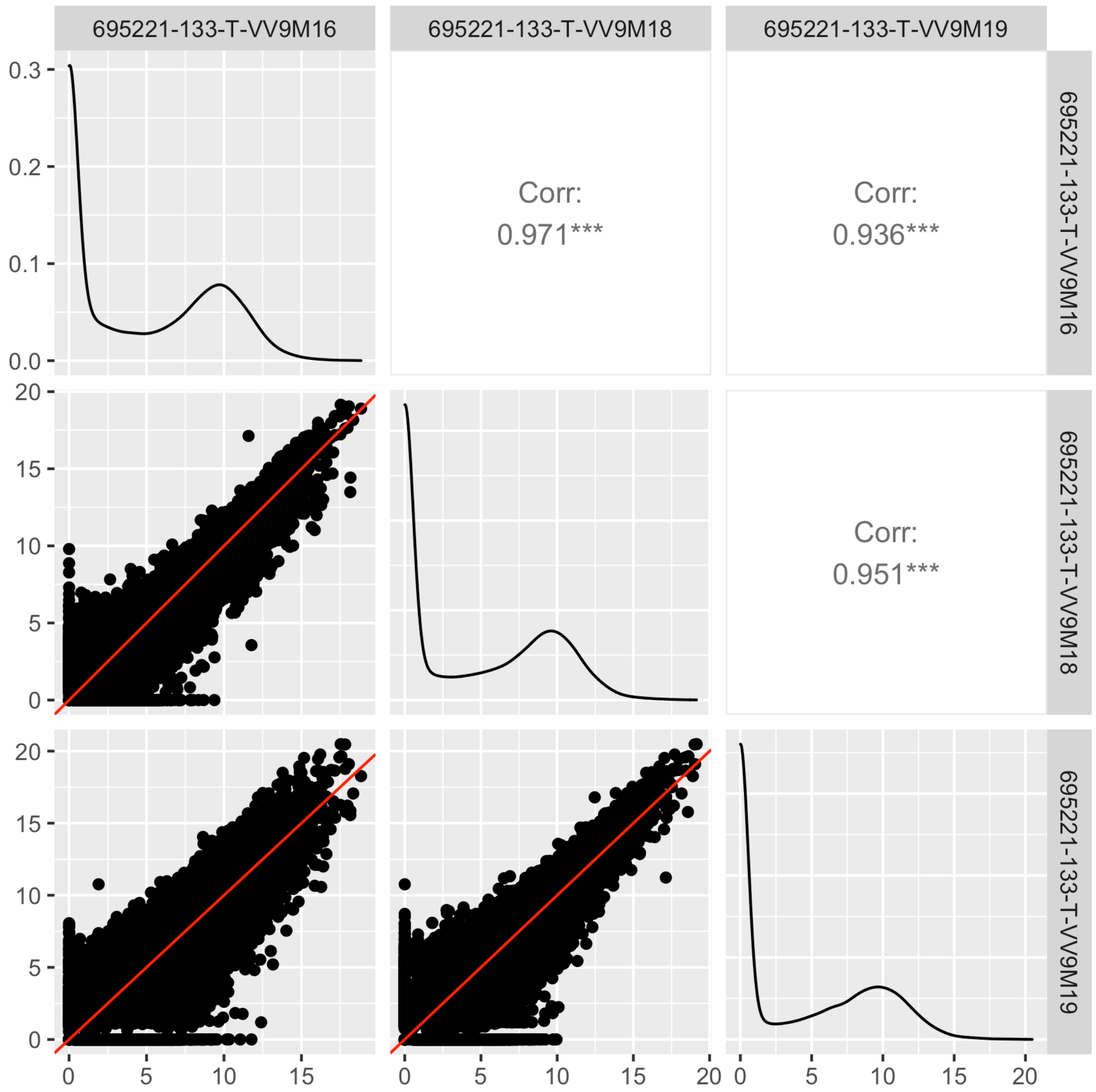

Figure S6A

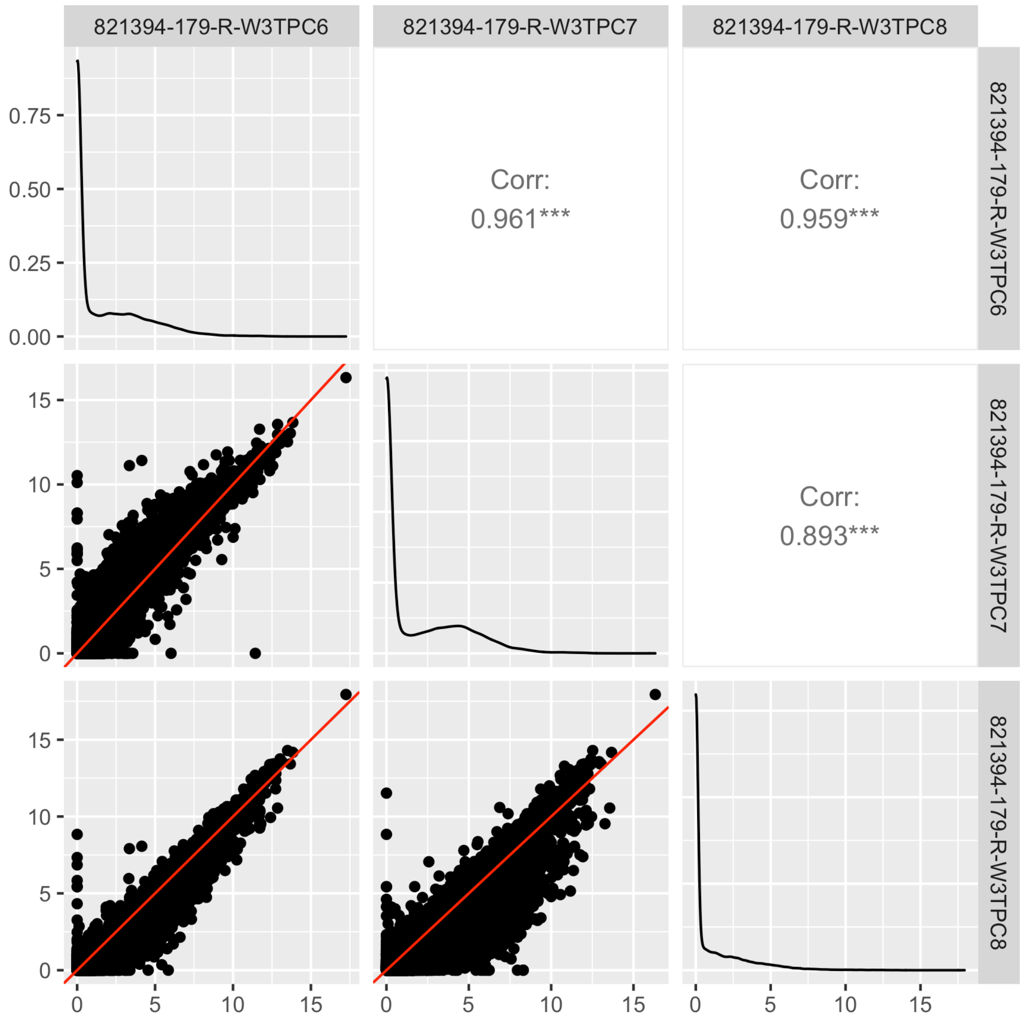

Figure S6B

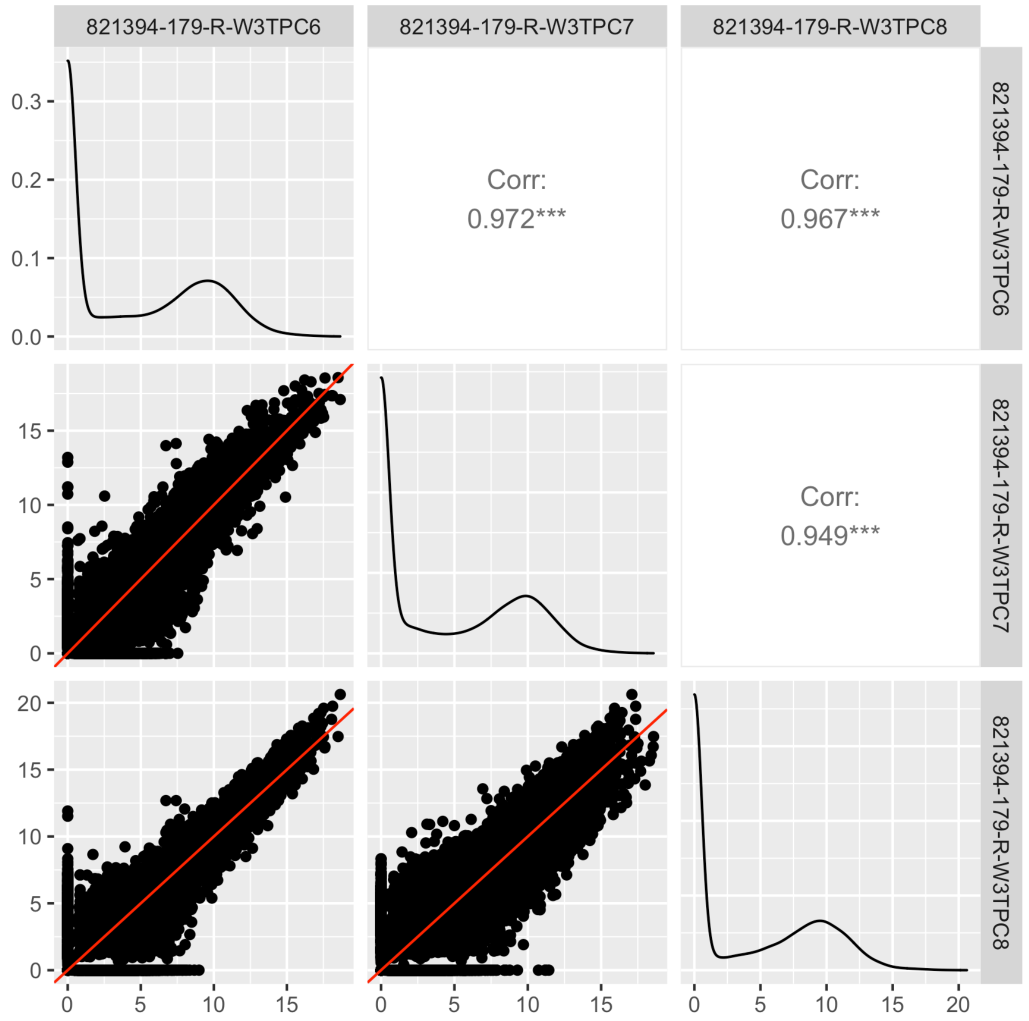

Figure S7A

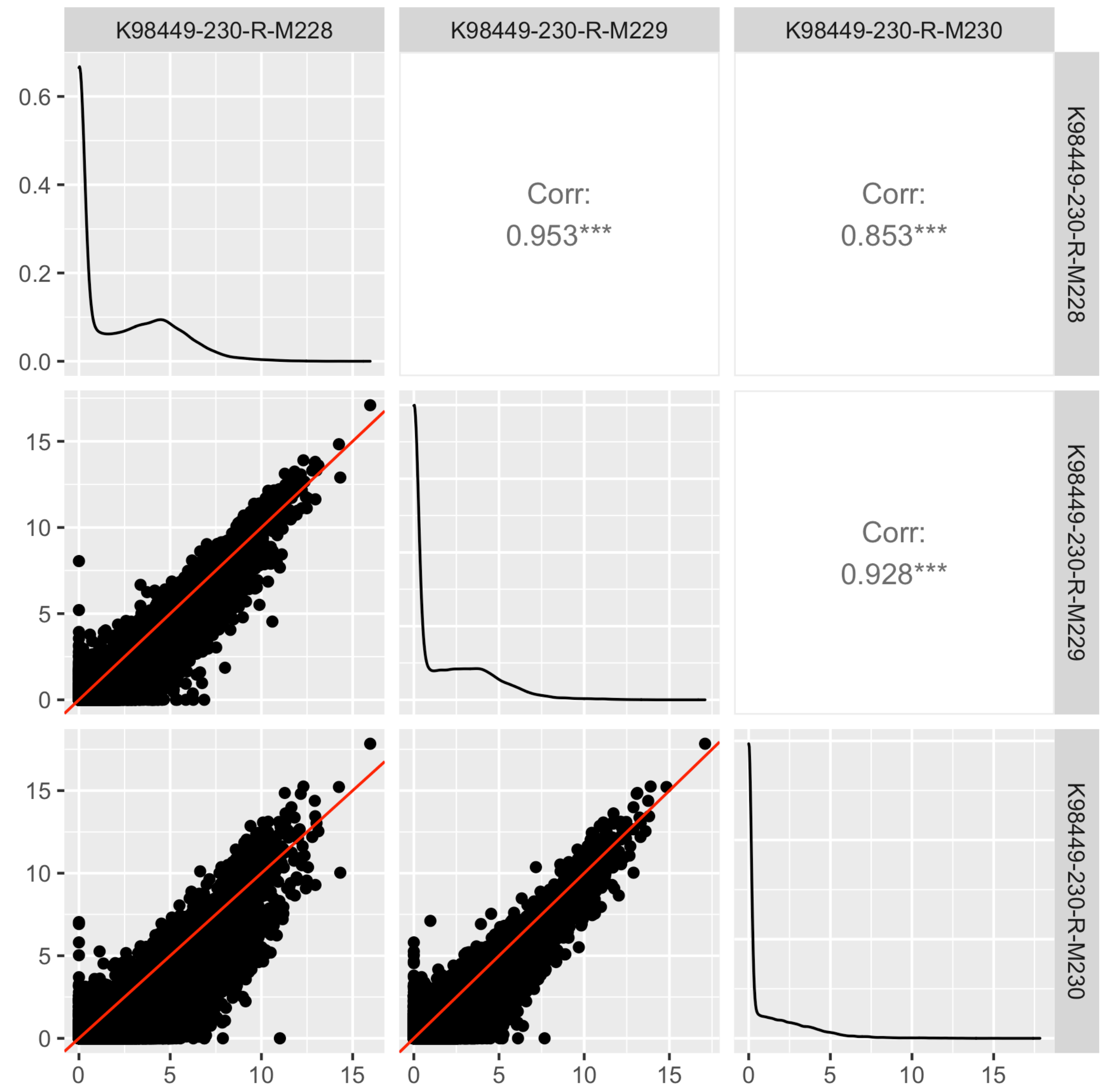

Figure S7B

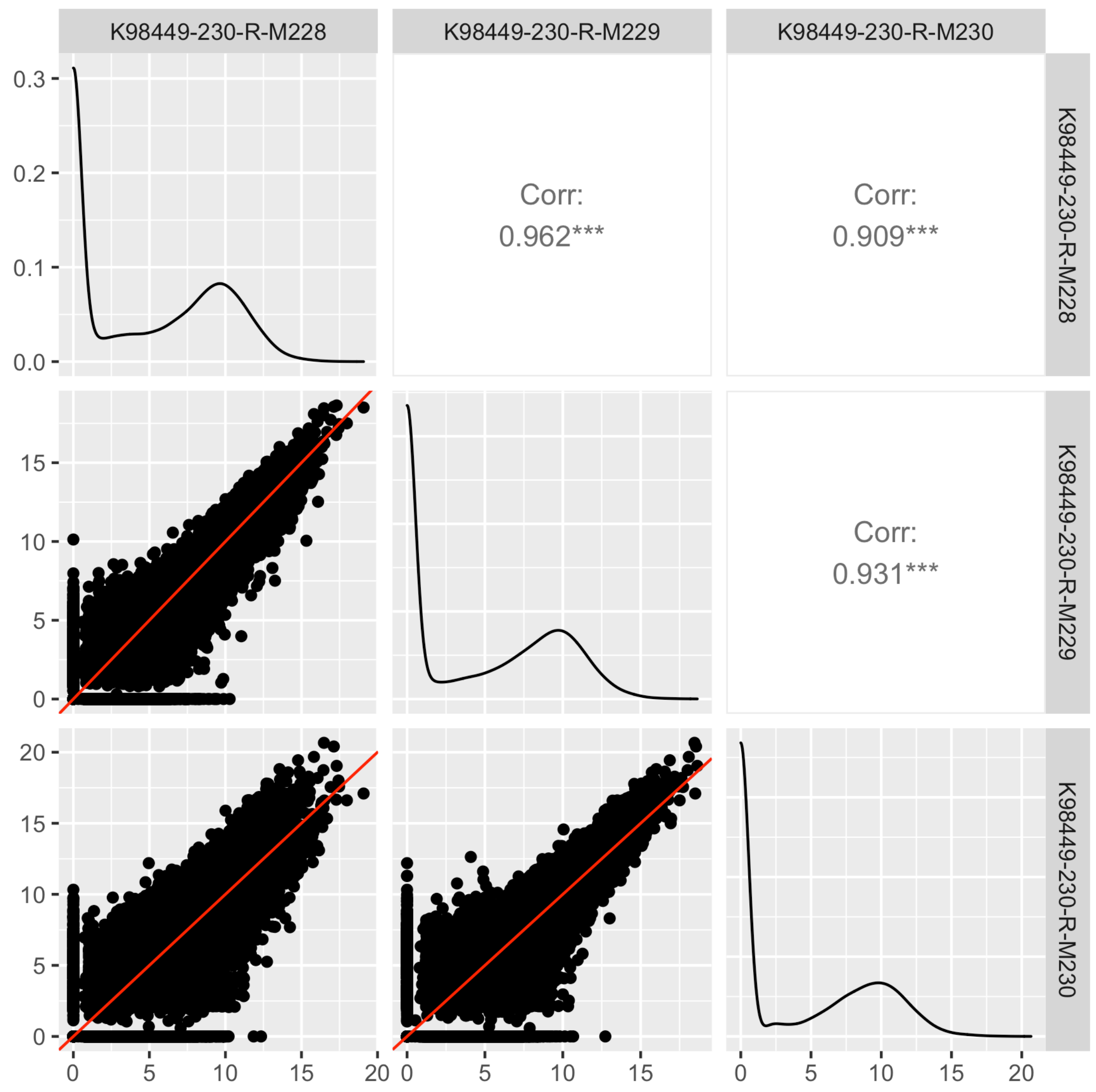

Figure S8A

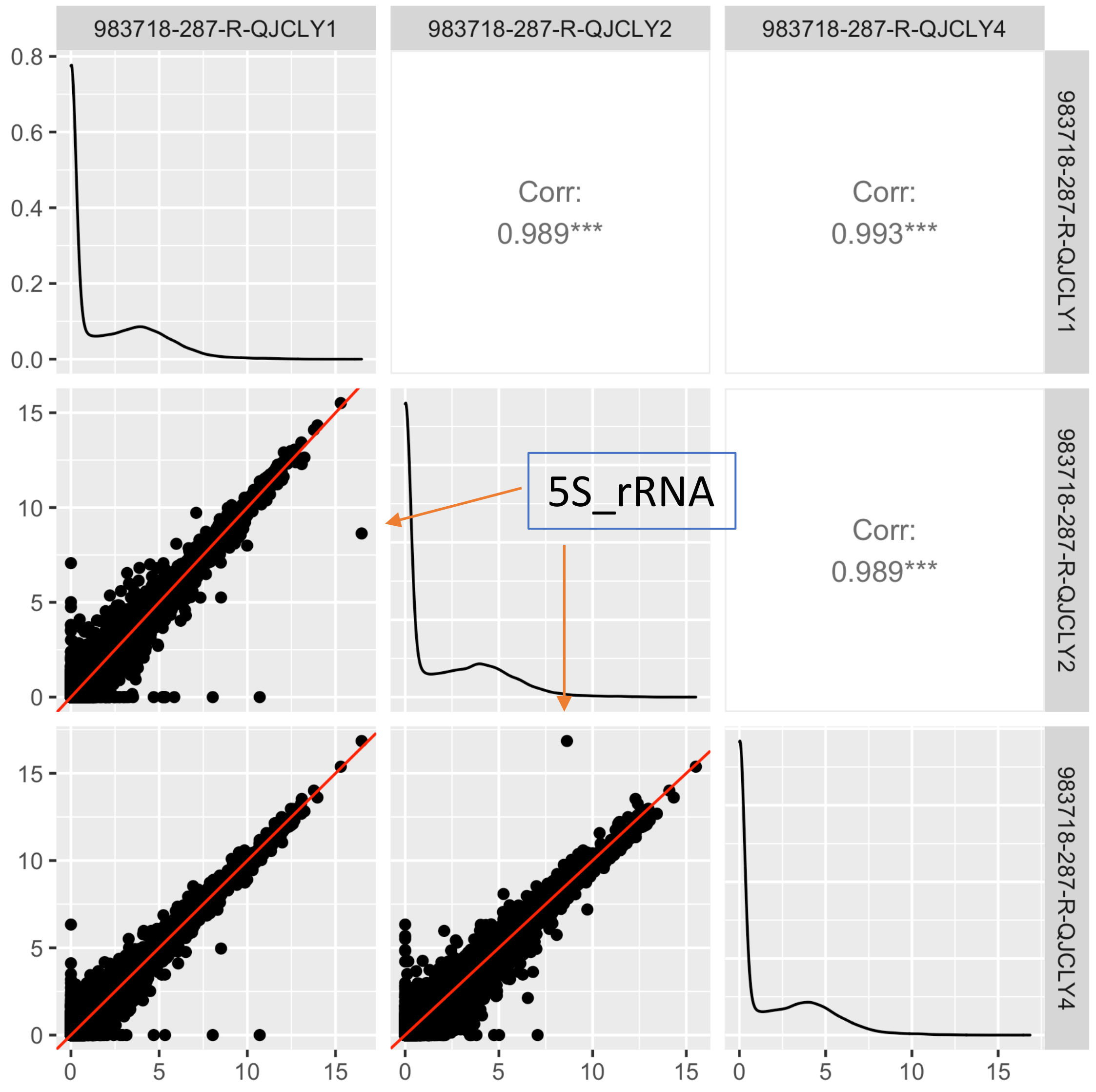

Figure S8B

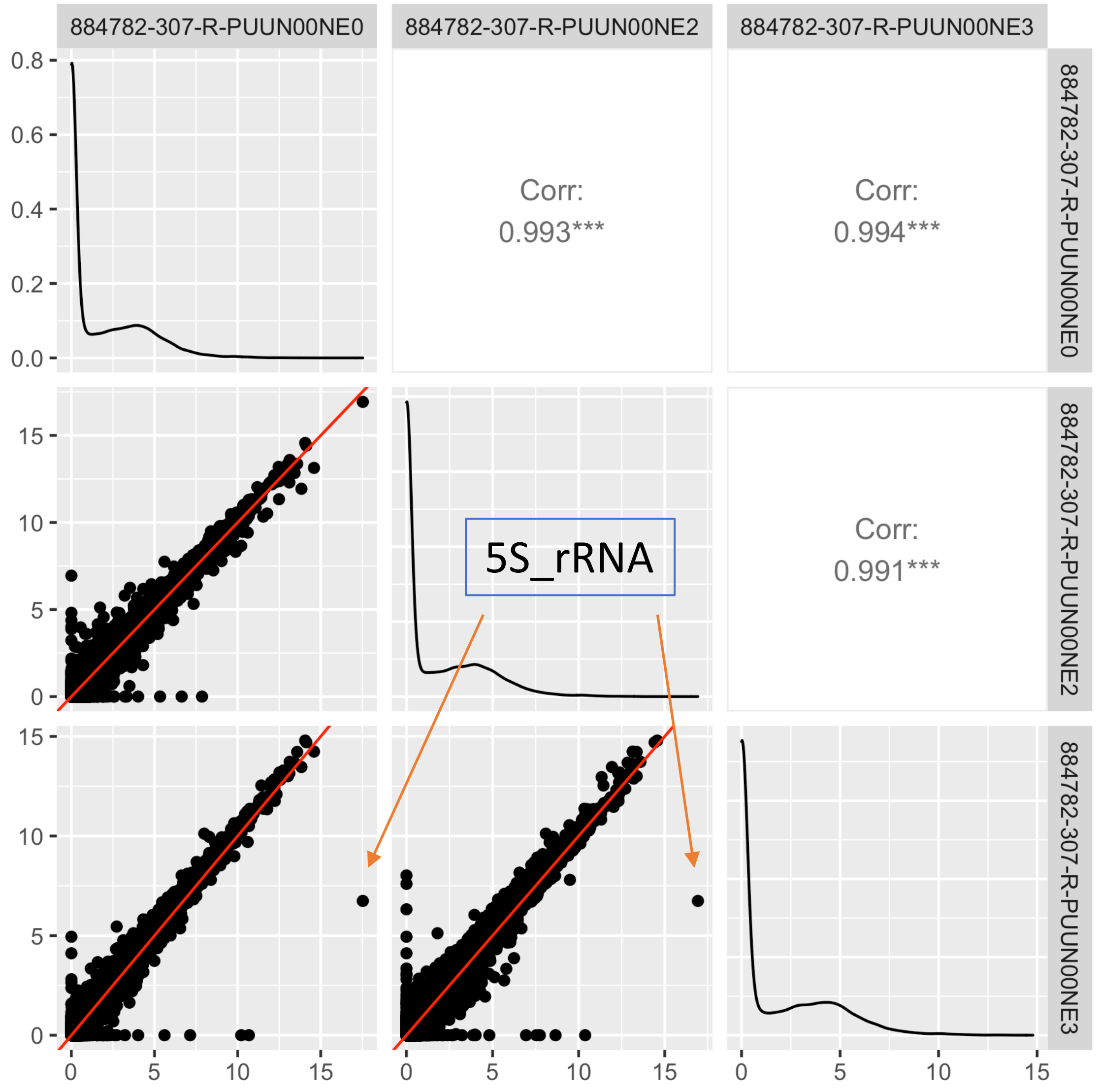

Table S1. Details of the patient-derived xenograft samples used in this study (downloaded on Sept. 16, 2020 from the NCI PDMR database).

EXPID	PatientID	SpecimenID	SampleID	Description	DiseaseBodyLocation
1	144126	210-T	JKQFX5	Neuroendocrine cancer, NOS	Endocrine and Neuroendocrine
2	144126	210-T	JKQFX6	Neuroendocrine cancer, NOS	Endocrine and Neuroendocrine
3	144126	210-T	JKQFX8	Neuroendocrine cancer, NOS	Endocrine and Neuroendocrine
4	159449	244-R	KNHY53B09	Transitional cell car. - uroth.	Genitourinary
5	159449	244-R	KNHY53B13	Transitional cell car. - uroth.	Genitourinary
6	159449	244-R	KNHY53B17	Transitional cell car. - uroth.	Genitourinary
7	272462	364-R	APP	RCC, clear cell adenocarcinoma	Genitourinary
8	272462	364-R	APQ	RCC, clear cell adenocarcinoma	Genitourinary
9	272462	364-R	APT	RCC, clear cell adenocarcinoma	Genitourinary
10	328469	098-R	E9CF81N88	Adenocarcinoma - colon	Digestive/Gastrointestinal
11	328469	098-R	E9CF81N89	Adenocarcinoma - colon	Digestive/Gastrointestinal
12	328469	098-R	E9CF81N91	Adenocarcinoma - colon	Digestive/Gastrointestinal
13	367327	171-R	WTFQQ4	Salivary gland cancer	Head and Neck
14	367327	171-R	WTFQQ6	Salivary gland cancer	Head and Neck
15	367327	171-R	WTFQQ7	Salivary gland cancer	Head and Neck
16	474343	323-R	AT8WB0	Osteosarcoma	Musculoskeletal
17	474343	323-R	AT8WB1	Osteosarcoma	Musculoskeletal
18	474343	323-R	AT8WB2	Osteosarcoma	Musculoskeletal
19	475296	252-R	KPNPN8	Adenocarcinoma - rectum	Digestive/Gastrointestinal
20	475296	252-R	KPNPN9	Adenocarcinoma - rectum	Digestive/Gastrointestinal
21	475296	252-R	KPNPP2	Adenocarcinoma - rectum	Digestive/Gastrointestinal
22	674366	145-R	HQKLK0	Non-Rhabdo. soft tissue sarcoma	Musculoskeletal
23	674366	145-R	HQKLK1	Non-Rhabdo. soft tissue sarcoma	Musculoskeletal
24	674366	145-R	HQKLK4	Non-Rhabdo. soft tissue sarcoma	Musculoskeletal
25	695221	133-T	VV9M16	Melanoma	Skin
26	695221	133-T	VV9M18	Melanoma	Skin
27	695221	133-T	VV9M19	Melanoma	Skin
28	697273	096-R	GYUQC3	Fibrosarcoma - not infantile	Musculoskeletal
29	697273	096-R	GYUQC4	Fibrosarcoma - not infantile	Musculoskeletal
30	697273	096-R	GYUQC6	Fibrosarcoma - not infantile	Musculoskeletal
31	791794	340-R	K7TB57	Squamous cervical cancer	Gynecologic

32	791794	340-R	K7TB58	Squamous cervical cancer	Gynecologic
33	791794	340-R	K7TB59	Squamous cervical cancer	Gynecologic
34	821394	179-R	W3TPC6	Malignant fibrous histiocytoma	Musculoskeletal
35	821394	179-R	W3TPC7	Malignant fibrous histiocytoma	Musculoskeletal
36	821394	179-R	W3TPC8	Malignant fibrous histiocytoma	Musculoskeletal
37	869496	051-R	UCEA97	Adenocarcinoma - rectum	Digestive/Gastrointestinal
38	869496	051-R	UCEA98	Adenocarcinoma - rectum	Digestive/Gastrointestinal
39	869496	051-R	UCEB05	Adenocarcinoma - rectum	Digestive/Gastrointestinal
40	884782	307-R	PUUN00NE0	Non-Rhabdo. soft tissue sarcoma	Musculoskeletal
41	884782	307-R	PUUN00NE2	Non-Rhabdo. soft tissue sarcoma	Musculoskeletal
42	884782	307-R	PUUN00NE3	Non-Rhabdo. soft tissue sarcoma	Musculoskeletal
43	945468	187-T	HAHVT8	Adenocarcinoma - rectum	Digestive/Gastrointestinal
44	945468	187-T	HAHVT9	Adenocarcinoma - rectum	Digestive/Gastrointestinal
45	945468	187-T	HAHVU5	Adenocarcinoma - rectum	Digestive/Gastrointestinal
46	947758	054-R	UCPB18	Adenocarcinoma - colon	Digestive/Gastrointestinal
47	947758	054-R	UCPB22	Adenocarcinoma - colon	Digestive/Gastrointestinal
48	947758	054-R	UCPB25	Adenocarcinoma - colon	Digestive/Gastrointestinal
49	947758	054-R	UCPB27	Adenocarcinoma - colon	Digestive/Gastrointestinal
50	952719	076-R	P53	Lung adenocarcinoma	Respiratory/Thoracic
51	952719	076-R	P56	Lung adenocarcinoma	Respiratory/Thoracic
52	952719	076-R	P57	Lung adenocarcinoma	Respiratory/Thoracic
53	983718	287-R	QJCLY1	Adenocarcinoma - colon	Digestive/Gastrointestinal
54	983718	287-R	QJCLY2	Adenocarcinoma - colon	Digestive/Gastrointestinal
55	983718	287-R	QJCLY4	Adenocarcinoma - colon	Digestive/Gastrointestinal
56	997726	040-R	X16	Squamous cell lung carcinoma	Respiratory/Thoracic
57	997726	040-R	X17	Squamous cell lung carcinoma	Respiratory/Thoracic
58	997726	040-R	X18	Squamous cell lung carcinoma	Respiratory/Thoracic
59	K98449	230-R	M228	Glioblastoma multiforme	Neurologic
60	K98449	230-R	M229	Glioblastoma multiforme	Neurologic
61	K98449	230-R	M230	Glioblastoma multiforme	Neurologic

Table S2. Summary statistics for CV values including interquartile range for different quantitative measures.

model	method	25	50	75	IQR
144126-210-T	COUNTDESEQ2	0.0388	0.0822	0.7792	0.7403
144126-210-T	COUNTTMM	0.0389	0.0805	0.7485	0.7096
144126-210-T	FPKM	0.0882	0.2246	0.9002	0.8119
144126-210-T	TPM	0.1049	0.2687	0.9043	0.7993
159449-244-R	COUNTDESEQ2	0.0317	0.0668	0.3207	0.289
159449-244-R	COUNTTMM	0.0311	0.0654	0.3146	0.2835
159449-244-R	FPKM	0.086	0.2006	0.7264	0.6404
159449-244-R	TPM	0.1231	0.263	0.7605	0.6374
272462-364-R	COUNTDESEQ2	0.0371	0.071	0.26	0.2229
272462-364-R	COUNTTMM	0.0361	0.0708	0.2553	0.2193
272462-364-R	FPKM	0.0894	0.2015	0.6533	0.5639
272462-364-R	TPM	0.0956	0.231	0.6801	0.5845
328469-098-R	COUNTDESEQ2	0.0149	0.032	0.1669	0.152
328469-098-R	COUNTTMM	0.0144	0.0308	0.1602	0.1458
328469-098-R	FPKM	0.0346	0.0818	0.4108	0.3761
328469-098-R	TPM	0.0364	0.0835	0.4074	0.371
367327-171-R	COUNTDESEQ2	0.0357	0.0701	0.2526	0.2168
367327-171-R	COUNTTMM	0.0352	0.0709	0.2495	0.2143
367327-171-R	FPKM	0.0887	0.2254	0.6241	0.5354
367327-171-R	TPM	0.1383	0.3166	0.6806	0.5423
474343-323-R	COUNTDESEQ2	0.0321	0.0619	0.2804	0.2483
474343-323-R	COUNTTMM	0.0315	0.0606	0.2721	0.2406
474343-323-R	FPKM	0.0765	0.1645	0.6113	0.5348
474343-323-R	TPM	0.0699	0.1765	0.6362	0.5663
475296-252-R	COUNTDESEQ2	0.0393	0.0796	0.2981	0.2588
475296-252-R	COUNTTMM	0.0383	0.0781	0.2888	0.2505
475296-252-R	FPKM	0.0873	0.2129	0.6867	0.5993
475296-252-R	TPM	0.1283	0.3324	0.8541	0.7259
674366-145-R	COUNTDESEQ2	0.0324	0.068	0.3453	0.313
674366-145-R	COUNTTMM	0.0309	0.0648	0.3332	0.3023
674366-145-R	FPKM	0.0767	0.1728	0.6533	0.5766
674366-145-R	TPM	0.0894	0.199	0.6672	0.5778
695221-133-T	COUNTDESEQ2	0.0618	0.134	0.8681	0.8063
695221-133-T	COUNTTMM	0.061	0.1331	0.8677	0.8067
695221-133-T	FPKM	0.1527	0.3867	0.9352	0.7824
695221-133-T	TPM	0.2195	0.5157	0.9984	0.7789
697273-096-R	COUNTDESEQ2	0.0203	0.0506	0.8666	0.8463
697273-096-R	COUNTTMM	0.02	0.0491	0.8663	0.8463
697273-096-R	FPKM	0.0549	0.1561	1.0467	0.9918
697273-096-R	TPM	0.0463	0.1261	0.9968	0.9504
791794-340-R	COUNTDESEQ2	0.0214	0.0445	0.2017	0.1803
791794-340-R	COUNTTMM	0.021	0.0437	0.1956	0.1746
791794-340-R	FPKM	0.0558	0.126	0.4886	0.4328
791794-340-R	TPM	0.0607	0.1443	0.4859	0.4252
821394-179-R	COUNTDESEQ2	0.0544	0.1105	0.4192	0.3648
821394-179-R	COUNTTMM	0.0545	0.1114	0.4174	0.3629
821394-179-R	FPKM	0.1346	0.3034	0.861	0.7264
821394-179-R	TPM	0.1906	0.4387	0.8898	0.6992
869496-051-R	COUNTDESEQ2	0.033	0.064	0.2138	0.1808

869496-051-R	COUNTTMM	0.0323	0.0628	0.211	0.1787
869496-051-R	FPKM	0.0758	0.1662	0.5519	0.4761
869496-051-R	TPM	0.101	0.2091	0.5897	0.4887
884782-307-R	COUNTDESEQ2	0.0143	0.0301	0.1917	0.1774
884782-307-R	COUNTTMM	0.0139	0.0291	0.1856	0.1716
884782-307-R	FPKM	0.039	0.0848	0.4377	0.3986
884782-307-R	TPM	0.0415	0.0866	0.4268	0.3852
945468-187-T	COUNTDESEQ2	0.0267	0.0636	0.5057	0.479
945468-187-T	COUNTTMM	0.0261	0.061	0.4902	0.4642
945468-187-T	FPKM	0.0759	0.2053	0.8735	0.7976
945468-187-T	TPM	0.1096	0.2587	0.8676	0.7579
947758-054-R	COUNTDESEQ2	0.0328	0.0691	0.415	0.3822
947758-054-R	COUNTTMM	0.0324	0.0681	0.4063	0.3739
947758-054-R	FPKM	0.0864	0.1907	0.731	0.6447
947758-054-R	TPM	0.0855	0.2052	0.7268	0.6413
952719-076-R	COUNTDESEQ2	0.0267	0.0557	0.2697	0.243
952719-076-R	COUNTTMM	0.0259	0.0535	0.2625	0.2367
952719-076-R	FPKM	0.0614	0.1413	0.5791	0.5177
952719-076-R	TPM	0.0662	0.1387	0.568	0.5018
983718-287-R	COUNTDESEQ2	0.0156	0.0353	0.1996	0.1839
983718-287-R	COUNTTMM	0.0151	0.0335	0.1919	0.1768
983718-287-R	FPKM	0.0367	0.0931	0.4723	0.4356
983718-287-R	TPM	0.0344	0.0846	0.4564	0.422
997726-040-R	COUNTDESEQ2	0.0473	0.0902	0.3186	0.2714
997726-040-R	COUNTTMM	0.047	0.0887	0.3092	0.2622
997726-040-R	FPKM	0.1134	0.2526	0.7312	0.6178
997726-040-R	TPM	0.1219	0.286	0.7639	0.642
K98449-230-R	COUNTDESEQ2	0.0597	0.124	0.8663	0.8066
K98449-230-R	COUNTTMM	0.0603	0.1251	0.8663	0.806
K98449-230-R	FPKM	0.1499	0.3389	0.9008	0.7509
K98449-230-R	TPM	0.2457	0.5125	0.9615	0.7158

Table S3A. The transcript percentages in each sample for the top five most abundant genes in four PDX models whose TPM data had the highest median CV values.

		Top 5 most highly expressed transcripts in model					
Model	***SampleID***	***RMRP***	***TMSB4X***	***SCARNA10***	***SCARNA2***	***SCARNA5***	***Total % TPM for top 5 genes***
K98449-230-R	M228	6.38	1.94	0.50	0.78	0.46	10.06
	M229	13.98	2.91	1.53	1.38	0.87	20.67
	M230	23.41	3.81	3.89	2.13	2.86	36.10
Model	***SampleID***	***RMRP***	***5S_rRNA***	***RPS17L***	***RPL9***	***TMSB4X***	***Total % TPM for top 5 genes***
475296-252-R	KPNPN8	11.45	1.66	3.21	2.07	1.48	19.89
	KPNPN9	4.57	2.39	1.86	0.94	0.94	10.69
	KPNPP2	8.98	4.13	2.31	1.85	1.35	18.62
Model	***SampleID***	***RMRP***	***RPS17L***	***RPL9***	***RPL19***	***UBB***	***Total % TPM for top 5 genes***
695221-133-T	VV9M16	10.55	3.15	0.69	1.28	1.01	16.68
	VV9M18	18.06	3.59	1.08	1.72	1.17	25.62
	VV9M19	22.62	3.67	2.24	1.00	0.96	30.48
Model	***SampleID***	***RMRP***	***Metazoa_SRP***	***RPL9***	***TMSB4X***	***RPS17L***	***Total % TPM for top 5 genes***
821394-179-R	W3TPC6	15.59	1.47	1.16	1.31	0.91	20.43
	W3TPC7	8.20	1.30	0.59	0.84	0.77	11.70
	W3TPC8	25.19	1.85	2.01	1.10	1.21	31.37

Table S3B. The transcript percentages in each sample for the top five most abundant genes in five PDX models whose TPM data had the lowest median CV values.

		Top 5 most highly expressed transcripts in model					
Model	***SampleID***	***RMRP***	***5S_rRNA***	***RPS17L***	***RPL9***	***ACTB***	***Total % TPM for top 5 genes***
328469-098-R	E9CF81N88	7.08	2.36	1.81	1.23	1.15	13.63
	E9CF81N89	7.27	3.28	2.17	1.49	0.97	15.18
	E9CF81N91	7.02	1.51	1.91	1.28	1.15	12.88
Model	***SampleID***	***5S_rRNA***	***RMRP***	***RPS17L***	***ACTB***	***RPL9***	***Total % TPM for top 5 genes***
983718-287-R	QJCLY1	9.14	4.02	1.61	1.40	0.86	17.03
	QJCLY2	0.04	4.67	2.06	1.75	1.11	9.63
	QJCLY4	11.81	4.26	1.25	1.65	0.66	19.64
Model	***SampleID***	***5S_rRNA***	***RMRP***	***ACTB***	***NBPF10***	***COL1A1***	***Total % TPM for top 5 genes***
884782-307-R	PUUN00NE0	18.92	1.71	1.80	2.47	1.22	26.12
	PUUN00NE2	12.45	2.42	2.18	0.90	1.06	19.01
	PUUN00NE3	0.01	2.84	2.67	1.93	1.91	9.35
Model	***SampleID***	***RMRP***	***HIST2H2AA4***	***UBB***	***HIST1H2BK***	***RPS17L***	***Total % TPM for top 5 genes***
952719-076-R	P53	11.04	1.70	1.24	1.17	1.04	16.20
	P56	7.24	1.86	1.29	1.14	0.93	12.46
	P57	12.68	1.11	0.64	0.77	1.08	16.28
Model	***SampleID***	***5S_rRNA***	***KRT6A***	***KRT5***	***RMRP***	***RPS17L***	***Total % TPM for top 5 genes***
791794-340-R	K7TB57	9.76	4.65	4.30	2.50	2.44	23.66
	K7TB58	11.72	4.92	4.71	2.57	2.34	26.26
	K7TB59	12.52	2.45	1.74	4.13	3.69	24.53
